# Unperturbed Cytotoxic Lymphocyte Phenotype and Function in Myalgic Encephalomyelitis/Chronic Fatigue Syndrome Patients

**DOI:** 10.3389/fimmu.2017.00723

**Published:** 2017-06-26

**Authors:** Jakob Theorell, Indre Bileviciute-Ljungar, Bianca Tesi, Heinrich Schlums, Mette Sophie Johnsgaard, Babak Asadi-Azarbaijani, Elin Bolle Strand, Yenan T. Bryceson

**Affiliations:** ^1^Department of Medicine, Huddinge, Karolinska Institutet, Stockholm, Sweden; ^2^Department of Rehabilitation Medicine, Karolinska Institutet, Stockholm, Sweden; ^3^Department of Clinical Sciences, Danderyd Hospital, Karolinska Institutet, Stockholm, Sweden; ^4^Childhood Cancer Research Unit, Department of Women’s and Children’s Health, Karolinska Institute, Karolinska University Hospital Solna, Stockholm, Sweden; ^5^Clinical Genetics Unit, Department of Molecular Medicine and Surgery, Center for Molecular Medicine, Karolinska Institute, Karolinska University Hospital Solna, Stockholm, Sweden; ^6^Balderklinikken, Oslo, Norway; ^7^Division of Medicine, CFS/ME Centre, Oslo University Hospital, Oslo, Norway; ^8^VID Specialized University, Oslo, Norway; ^9^Norwegian National Advisory Unit on CFS/ME, Oslo University Hospital, Oslo, Norway; ^10^Department of Clinical Science, University of Bergen, Bergen, Norway

**Keywords:** chronic fatigue syndrome, myalgic encephalomyelitis, natural killer cells, cytotoxic T cells, lymphocyte cytotoxicity, adaptive natural killer cells

## Abstract

Myalgic encephalomyelitis or chronic fatigue syndrome (ME/CFS) is a debilitating disorder linked to diverse intracellular infections as well as physiological stress. Cytotoxic lymphocytes combat intracellular infections. Their function is attenuated by stress. Despite numerous studies, the role of cytotoxic lymphocytes in ME/CFS remains unclear. Prompted by advances in the understanding of defects in lymphocyte cytotoxicity, the discovery of adaptive natural killer (NK) cell subsets associated with certain viral infections, and compelling links between stress, adrenaline, and cytotoxic lymphocyte function, we reassessed the role of cytotoxic lymphocytes in ME/CFS. Forty-eight patients from two independent cohorts fulfilling the Canada 2003 criteria for ME/CFS were evaluated with respect to cytotoxic lymphocyte phenotype and function. Results were compared to values from matched healthy controls. Reproducible differences between patients and controls were not found in cytotoxic lymphocyte numbers, cytotoxic granule content, activation status, exocytotic capacity, target cell killing, or cytokine production. One patient expressed low levels of perforin, explained by homozygosity for the *PRF1* p.A91V variant. However, overall, this variant was present in a heterozygous state at the expected population frequency among ME/CFS patients. No single patient displayed any pathological patterns of cellular responses. Increased expansions of adaptive NK cells or deviant cytotoxic lymphocyte adrenaline-mediated inhibition were not observed. In addition, supervised dimensionality reduction analyses of the full, multidimensional datasets did not reveal any reproducible patient/control discriminators. In summary, employing sensitive assays and analyses for quantification of cytotoxic lymphocyte differentiation and function, cytotoxicity lymphocyte aberrances were not found among ME/CFS patients. These assessments of cytotoxic lymphocytes therefore do not provide useful biomarkers for the diagnosis of ME/CFS.

## Introduction

Myalgic encephalomyelitis or chronic fatigue syndrome (ME/CFS) is a heterogeneous disorder of unknown etiology. Different sets of criteria have been applied to diagnose the disorder (1–4). In the most recent update of the clinical consensus criteria, ME/CFS is characterized by flares of pronounced physical and/or cognitive fatigability and exacerbation of flu-like symptoms in response to exertion, which is combined with different neurological, immunological, and gastrointestinal symptoms that often fluctuate in severity (4). These debilitating symptoms reduce the patient’s ability to perform daily activities with at least 50% from premorbid level over an extended period of time, typically at least 6 months (4).

Several epidemics of debilitating, long-lasting fatigue have been reported, occasionally associated with specific infections (5–7). Population-based investigations in the United Kingdom have estimated a stable yearly incidence of ME/CFS to 0.015% of the population, with a prevalence of approximately 0.1% (8). High antibody titers to the herpes viruses such as cytomegalovirus (CMV) and Epstein–Barr virus (EBV) in a group of patients with a chronic mononucleosis-like phenotype provided initial links between infection and ME/CFS (9). Infections by other intracellular pathogens have also been proposed to trigger the disorder (10). A prospective study of patients initially presenting with acute infection with EBV or two other intracellular pathogens at primary health-care centers found that, 6 months postinfection, 11% of the patients fulfilled ME/CFS criteria, regardless of infectious agent (11). In that study, the risk for development of ME/CFS specifically correlated to the severity of the acute illness. Together, these observations suggest that impaired immunity to intracellular pathogens may be associated with ME/CFS susceptibility.

Generally referred to as cytotoxic lymphocytes, cytotoxic T lymphocytes (CTL) and natural killer (NK) cells use complementary strategies to protect the host from intracellular pathogens. They use somatically recombined or germ line-encoded receptors, respectively, for identification of infected cells, but share a molecular machinery for target cell killing. In an examination of 41 ME/CFS patients, Caligiuri et al. reported a decreased frequency of peripheral blood CD3^−^CD57^+^ lymphocytes, primarily representing NK cells, whereas frequencies of CD3^+^CD57^+^ cells, representing CTL, were unperturbed (12). Moreover, 73% of the patients displayed low target cell killing, as measured by lysis of K562 target cells (12). A number of studies have subsequently reported differences in the number, frequency, and function of peripheral blood NK cells from ME/CFS patients (13–15). However, other studies, including one study of 31 healthy or ME/CFS discordant monozygotic twin pairs, have not found such impairments (16–18). Thus, no consensus has been reached regarding a link between impaired lymphocyte cytotoxicity and ME/CFS. Moreover, possible mechanisms remain unclear.

Underscoring a pivotal role of cytotoxic lymphocytes in immunity and control of inflammation, genetic mutations impairing lymphocyte cytotoxicity are associated with severe diseases (19, 20). Whereas autosomal recessive nonsense mutations cause early-onset, life-threatening hyperinflammatory disorders, milder missense mutations are associated with a range of diseases encompassing hematologic malignancies, systemic autoimmunity, or severe neurological manifestations (19). Assessment of cellular function and protein levels, rather than DNA sequencing, offers a more integrated diagnostic approach to finding impairments in cytotoxic lymphocyte function. Sequencing may fail to identify mutations in non-coding, regulatory elements of genes (21). Technological advances have yielded diagnostic assays with high sensitivity and specificity for defects in lymphocyte cytotoxicity (22, 23). These assays are better suited than previous assays to identify patients with partial, primary defects in lymphocyte cytotoxicity, which hypothetically may be linked to ME/CFS development.

Besides primary defects in lymphocyte cytotoxicity, environmental factors may also influence cytotoxic lymphocyte function. Studies have recently uncovered that NK cells with unique, epigenetic adaptive features emerge in a subgroup of individuals upon infection with CMV (24, 25), which 60–70% of the population is infected by. Adaptive NK cells can persist independently of canonical NK cells and display reduced immunoregulatory capacity, thus being devoted to combating infection (26, 27). Coinfection with EBV potentiates the effect of CMV on cytotoxic lymphocyte differentiation (28). Large expansions of adaptive, non-immunoregulatory NK cells could thereby provide a link between certain herpes virus infections, excessive inflammation, and development of ME/CFS. Acute stress, another environmental factor, functionally inhibits cytotoxic lymphocytes in general and NK cells in particular. This process is a catecholamine-dependent effect mediated through the β2-adrenergic receptor (29–31). In addition to viral infections, acute physical and/or psychological stress can promote ME/CFS flares (4). An altered response to acute stress, potentially driven by aberrant β2-adrenergic receptors signaling could thus also lead to excessive inflammation and thence predispose to ME/CFS.

With this background, extensive analyses of peripheral blood cytotoxic lymphocyte phenotype and function in ME/CFS patients as compared to matched controls from two independent centers were performed, aiming to elucidate whether aberrances in lymphocyte cytotoxicity may be associated with ME/CFS.

## Materials and Methods

### Patients and Controls

The study was approved by regional Ethical Review Boards in Stockholm (EPN Stockholm) and Oslo (REK Sør-Øst C). The Swedish patients were recruited at the ME/CFS Rehabilitation Unit, Danderyd University Hospital, Stockholm. Controls were age and sex matched to the patients for each substudy individually. The Norwegian patients were recruited at the CFS/ME Centre, Oslo University Hospital, Oslo. Written informed consent was obtained from all patients as well as from the healthy controls recruited from the Oslo University Hospital Blood Bank. Oral informed consent was obtained from healthy controls recruited from the Stockholm Blood Bank (for which only sex and age is known). All patients fulfilled the Canada criteria for ME/CFS (2). Samples from patients were collected at steady state of the disorder.

### Cells

From the patients in the Stockholm substudy, peripheral blood was collected in heparin tubes and processed within 8 h of venipuncture. Whole blood used for TruCount analyses was obtained at this step. Peripheral blood mononuclear cells (PBMC) were obtained by density gradient centrifugation (Lymphoprep, Axis-Shield, Dundee, Scotland), washed twice in PBS, and immediately frozen down in fetal bovine serum (FBS) supplemented with 10% dimethylsulfoxide (DMSO, Sigma-Aldrich, St. Louis, MO, USA). For the Stockholm controls, blood was collected in the blood bank. During blood harvest, a heparinized blood sample was collected and used for TruCount analyses. The harvested blood bag was subsequently centrifuged in the blood bank, and a primary buffy coat was obtained. This buffy coat was subsequently further purified to PBMC by density gradient centrifugation, washed twice in PBS, and immediately frozen down in FBS supplemented with 10% DMSO. All this was done within 10 h of venipuncture.

In the Oslo substudy, peripheral blood from both patients and controls was collected in CPT tubes and processed within 2.5 h of venipuncture. PBMC were obtained by centrifugation of the CPT tubes, and the pellets were immediately frozen down in a freezing medium containing 45% FCS, 45% Roswell Parks Memorial Institute (RPMI), and 10% DMSO.

For phenotypic analyses, the cells were gently thawed the day of use directly in ice-cold FACS buffer [PBS with 2% FCS and 2 mM ethylenediaminetetraacetic acid (Sigma-Aldrich)] and counted using a Muse counter (Merck Millipore, Billerica, MA, USA) and an LSR Fortessa (BD Biosciences, Franklin Lakes, NJ, USA) for the Stockholm and Oslo samples, respectively.

For functional analyses, the cells were gently thawed in RPMI 1640 media, counted using a Muse counter (Merck Millipore) or an LSR Fortessa (BD Biosciences), and transferred to complete media [RPMI 1640 supplemented with 10% FBS and 1 mM l-glutamine (Hyclone, Logan, UT, USA)] at the approximate concentration of 3 × 10^6^ cells/ml. In the case of the Oslo T cell stimulations, 1 U DNAse-I (recombinant, Roche, Penzberg, Germany) was added, to prevent clotting of cells. All cells were subsequently rested at 37°C with 5% CO_2_ in a humified incubator until the next day when experiments were conducted.

For evaluation of exocytosis, three cell lines were used. K562 is an MHC class 1-deficient erythroleukemia cell line highly sensitive to NK cell killing, Raji is an EBV-transformed B-cell line that weakly triggers NK cell cytotoxicity. P815 is a mouse mastocytoma cell line expressing Fc receptors and hence can be coated with mouse antibodies specific to human antigens to induce redirected lysis. All cell lines were kept in complete media and were in logarithmic growth phase when used.

### General Experimental Design

All experiments, except for the TruCount, were conducted in one session. As such, the stainings were simultaneously conducted on PBMC thawed from all individuals from one study site (Stockholm or Oslo), eliminating inter-experimental variability. Details regarding specific assay protocols are provided in the Section “Supplementary Methods” in Supplementary Material.

### Flow Cytometry

All flow cytometry was performed on 18-color LSR Fortessa instruments (BD Biosciences). Subsequent flow cytometry file analysis was performed with FlowJo (version 9.8.1, TreeStar Inc., Ashland, OR, USA). For all flow cytometry assays, fluorochrome-conjugated antibodies have been used. See Table S1 in Supplementary Material for specifications. In addition to the specified antibodies, a fixable dead cell marker (Invitrogen) was used in all stainings. See the Section “Supplementary Methods” in Supplementary Material for staining protocols and gating strategies.

### Genetic Analyses

Genomic DNA was isolated from peripheral blood according to standard procedures. For the patient with low perforin expression, the coding regions of *PRF1* (NM_001083116.1, GRCH37) were amplified and sequenced on an ABI 3730 Genetic Analyzer (Thermo Fisher Scientific, Waltham, MA, USA). SeqScape (Version 2.5; Applied Biosystems) was used for analysis.

For genotyping of the *PRF1* p.A91V polymorphism (rs35947132), a validated TaqMan genotyping assay (c_25600964_20; Thermo Fisher Scientific) was used according to the manufacture’s instructions. Reactions were performed in duplicates, with positive (heterozygous and homozygous for the polymorphism) and negative controls included in each experiment. End point fluorescence detection was performed post-PCR on a QuantStudio 7 Flex Real-Time PCR System (Life Technologies, Thermo Fisher Scientific). Results were analyzed with the QuantStudio 6 and 7 Flex Software.

### Statistical Analyses

To decrease α-error rates due to multiple testing, only findings with *p*-values less than 0.01 are reported in the manuscript. Further, only values meeting the Holm–Bonferroni (32) correction for multiple testing are reported as significant. The Holm–Bonferroni correction was applied separately to each subfigure. To decrease β-error rates, the chosen α-error rate for the Holm–Bonferroni corrected *p*-values was 0.1, as all findings in the Stockholm substudy were confirmed in the Oslo substudy, and thus a value with *p* = 0.1 in both studies equals 0.1 × 0.1 = 0.01. Specific descriptions on statistical procedures, including data preprocessing, principal component (PC), and sparse partial least squares discriminant analyses, are described in the Section “Supplementary Methods” in Supplementary Material.

### Graphical Layouts

Tables have been generated using Excel software (Microsoft, version 14.4.9, Redmond, WA, USA). Figures have been generated using Prism (version 6.0b, GraphPad, La Jolla, CA, USA), R [version 3.2 (33)] including package ggplot2 (34) and gplots (35) and Illustrator (version 16.0.0, Adobe, San Jose, CA, USA).

## Results

### Patient and Control Characteristics

All 48 patients fulfilled Canada 2003 (2) criteria for ME/CFS. The clinical characteristics of the groups are summarized in Table 1 and Table S1 in Supplementary Material. The healthy control and patient groups were matched in terms of age and gender. In the Stockholm and Oslo patient groups, respectively, 100 and 88% of the patients suffered from ME/CFS for more than 2 years. An association between infection or vaccination and ME/CFS debut was noted in 83 and 96% of the cases from Stockholm and Oslo, respectively.

**Table 1:** Participant characteristics.

Sample	Group	Number of participants	Age		Females, %	Duration >2 years, %	Infectious trigger, %	Cytomegalovirus positive (*n* analyzed)
			Median	Range				
Stockholm	Patient	24	44	34–63	75	100	83	53 (17)
	Control	28	44	24–65	71	–	–	64 (28)
Oslo	Patient	24	30	18–52	79	88	96	
	Control	24	30	20–46	79	–	–	

### Evaluation of Peripheral Blood Cytotoxic Lymphocyte Numbers and Activation Status

**Figure 1:**
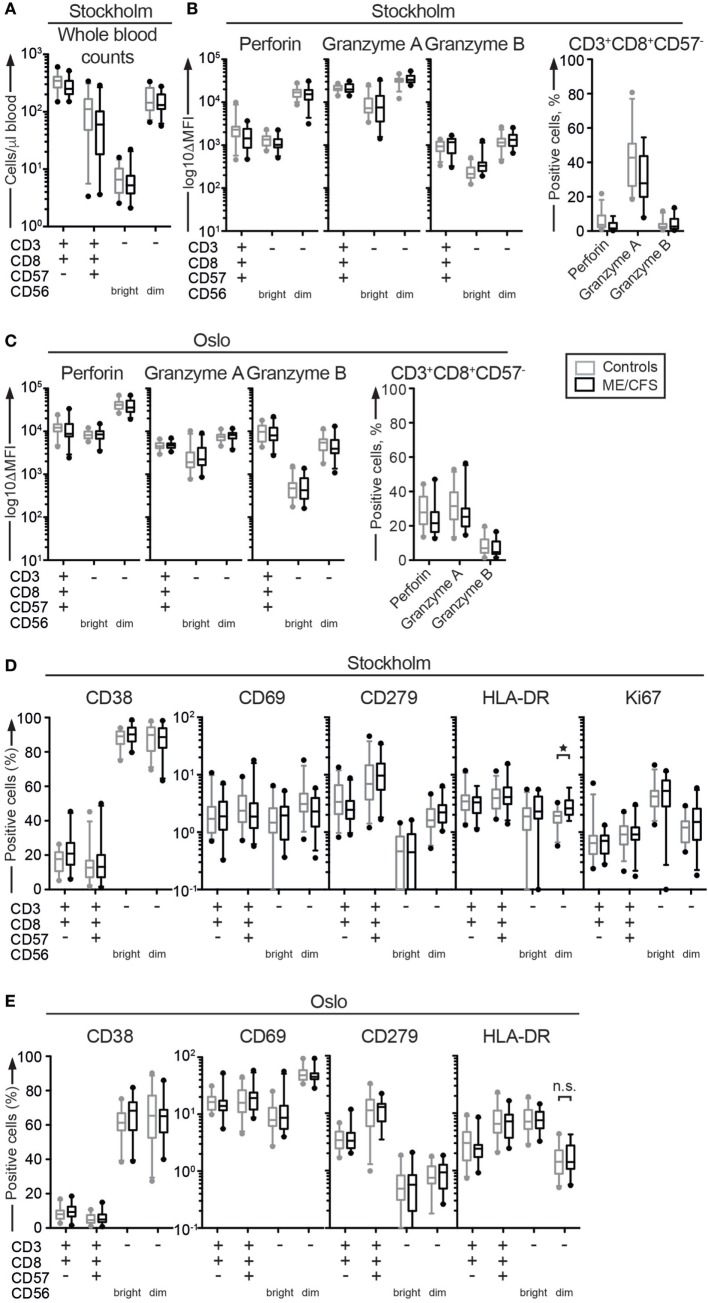
Cytotoxic lymphocyte counts, granule content, or activation status do not differ between ME/CFS patients and controls. **(A)** Absolute count of cytotoxic lymphocytes in whole blood from the Stockholm substudy. Whole blood was stained in tubes with predefined numbers of beads, and the bead-cell mix was analyzed with flow cytometry. Four CD8^+^ T cell and natural killer (NK) cell subsets are shown. **(B,C)** Phenotypic analysis of cytotoxic proteins in cytotoxic lymphocytes. Peripheral blood mononuclear cells (PBMC) were thawed, counted, and stained with antibodies to cytotoxic proteins and lineage markers. The level of perforin and granzyme A and B protein expression in four CD8^+^ T cell and NK cell subsets from Stockholm and Oslo is shown. ΔMFI = median fluorescence intensity after subtraction of median fluorescence intensity for isotype controls. CD57^−^CD8^+^ T cells are shown separately, as the cytotoxic proteins are expressed bimodally in these cells. **(D,E)** Phenotypic analysis of activation and mitosis status in cytotoxic lymphocytes. PBMC were thawed, counted, and stained with antibodies to activation and mitosis markers as well as lineage markers. **(D)** Percentage of four CD8^+^ T cell and NK cell subsets from the Stockholm substudy that are positive for the activation markers CD38, CD69, CD279, and HLA-DR and the mitosis marker Ki67. **(E)** Percentage of four CD8^+^ T cell and NK cell subsets from the Oslo substudy that are positive for the activation markers CD38, CD69, CD279, and HLA-DR. Gray boxes depict control values, whereas black boxes depict patient values. Lines through boxes show the median. Error bars extend to 5th and 95th percentile. Dots show outliers. 24 patients and 28 controls for the Stockholm substudy and 24 patients and 24 controls [20 patients and 23 controls for panel **(E)**] for the Oslo substudy are included in the analyses.

In an effort to acquire possible evidence for primary defects in lymphocyte cytotoxicity underlying ME/CFS, we wished to evaluate multiple aspects of cytotoxic lymphocyte function. A prerequisite for lymphocyte cytotoxicity is normal development and survival of cytotoxic lymphocyte subsets. Studies of ME/CFS patients have described decreased numbers of certain cytotoxic lymphocyte subsets (12, 13). Thus, we examined the numbers of four CD8^+^ T cell and NK cell subsets in the peripheral blood of ME/CFS patients. Among CD8^+^ T cells, CD3^+^CD4^−^CD8^+^CD57^−^ cells represent predominately non-cytotoxic subsets, whereas CD3^+^CD4^−^CD8^+^CD57^+^ T cells are *bona fide* cytotoxic effectors (23, 36). Peripheral blood NK cells can be grossly divided into CD3^−^CD56^bright^ immunoregulatory NK cells and CD3^−^CD56^dim^ NK cells with a strong cytotoxic capacity. Whole blood from patients and controls from Stockholm was collected, stained within 6 h of venipuncture, and analyzed by flow cytometry. No significant differences between patients and controls were identified with respect to the absolute numbers of peripheral blood CD8^+^ T cell or NK cell subsets (Figure 1A). Peripheral blood cell counts were not assessed in the Oslo substudy.

Previous reports have also described low levels of perforin in NK cell subsets (37). Congenital perforin deficiency results in fatal, infantile hyperinflammatory disorders (38). Granzyme A and B facilitate perforin-dependent cytotoxicity, diffusing into the target cell through pores formed by perforin and inducing apoptosis through protease activity. Although no patients with *GZMA* or *GZMB* mutations have so far been identified, *Gzma* and *Gzmb* double knockout mice develop hyperinflammatory disease upon viral infection (38). Expression levels of these cytotoxic granule constituents were therefore determined in CD8^+^ T cell and NK cell subsets from ME/CFS patients. PBMC were thawed, surface stained with antibodies to lineage markers, fixed, permeabilized, and stained intracellularly with antibodies to perforin, granzyme A, and granzyme B. Cells were analyzed by flow cytometry. Contrasting some previous reports, no systematic differences were identified with respect to cellular content of perforin (Figure 1B). One patient did, however, express low levels of perforin in CD8^+^ T cell and NK cell subsets. In this patient, sequencing revealed homozygosity for the *PRF1* p.A91V variant. When screening through all 24 Norwegian and 17 of the Swedish patient samples for which DNA was available, an additional three Swedish patients and one Norwegian patient were heterozygous for this *PRF1* variant. Thus, the carriership of the *PRF1* p.A91V variant among the analyzed patients (minor allele frequency 6/82 alleles, i.e., 7.3%), was comparable to that of >30,000 individuals in the European Exome Aggregation Consortium (minor allele frequency 4.7%) (39).

No differences were detected in regards to granzyme B expression among the cytotoxic CD8^+^ T cell or NK cell subsets from the Stockholm or Oslo patient samples (Figure 1B). However, in the non-cytotoxic CD8^+^CD57^−^ T cells, granzyme A levels were lower among the ME/CFS patients from Stockholm as compared to controls (*p* = 0.005, not significant after correction for multiple testing). There was a similar, insignificant tendency in the Oslo sample (*p* = 0.14) (Figure 1C).

Studies of ME/CFS patients have found increased CD69 expression on NK cells (40), decreased CD38 (40) or, by comparison to MS patients, increased CD38 and HLA-DR expression on CD8^+^ T cells (41), suggesting aberrant activation of cytotoxic lymphocytes in ME/CFS patients. Cell proliferation, measured by Ki67, has also been reported to be low, albeit selectively in CD4^+^ T cells (40). Thus, we examined expression of activation and proliferation markers in the four CD8^+^ T and NK cell subsets. PBMC were thawed, stained with antibodies to lineage markers as well as CD38, CD69, CD279 (PD-1), and HLA-DR, fixed, permeabilized, and stained intracellularly with an antibody to Ki67. Cells were analyzed by flow cytometry. In the patients from Stockholm, HLA-DR levels were higher on CD56^dim^ NK cells from the ME/CFS patients compared to controls (*p* = 0.002, significant after correction for multiple testing) (Figure 1D). No other differences were noted. In the Oslo substudy, the HLA-DR levels on CD56^dim^ NK cells were equal between the groups (*p* = 0.52), and no other differences were noted (Figure 1E). In summary, the ME/CFS patients exhibited normal numbers of cytotoxic lymphocytes that displayed normal cytotoxic protein content overall normal activation status. A non-significant trend for decreased granzyme A was noted in non-cytotoxic CD8^+^CD57^−^ T cells from ME/CFS patients.

### Evaluation of Peripheral Blood Cytotoxic Lymphocyte Function

**Figure 2:**
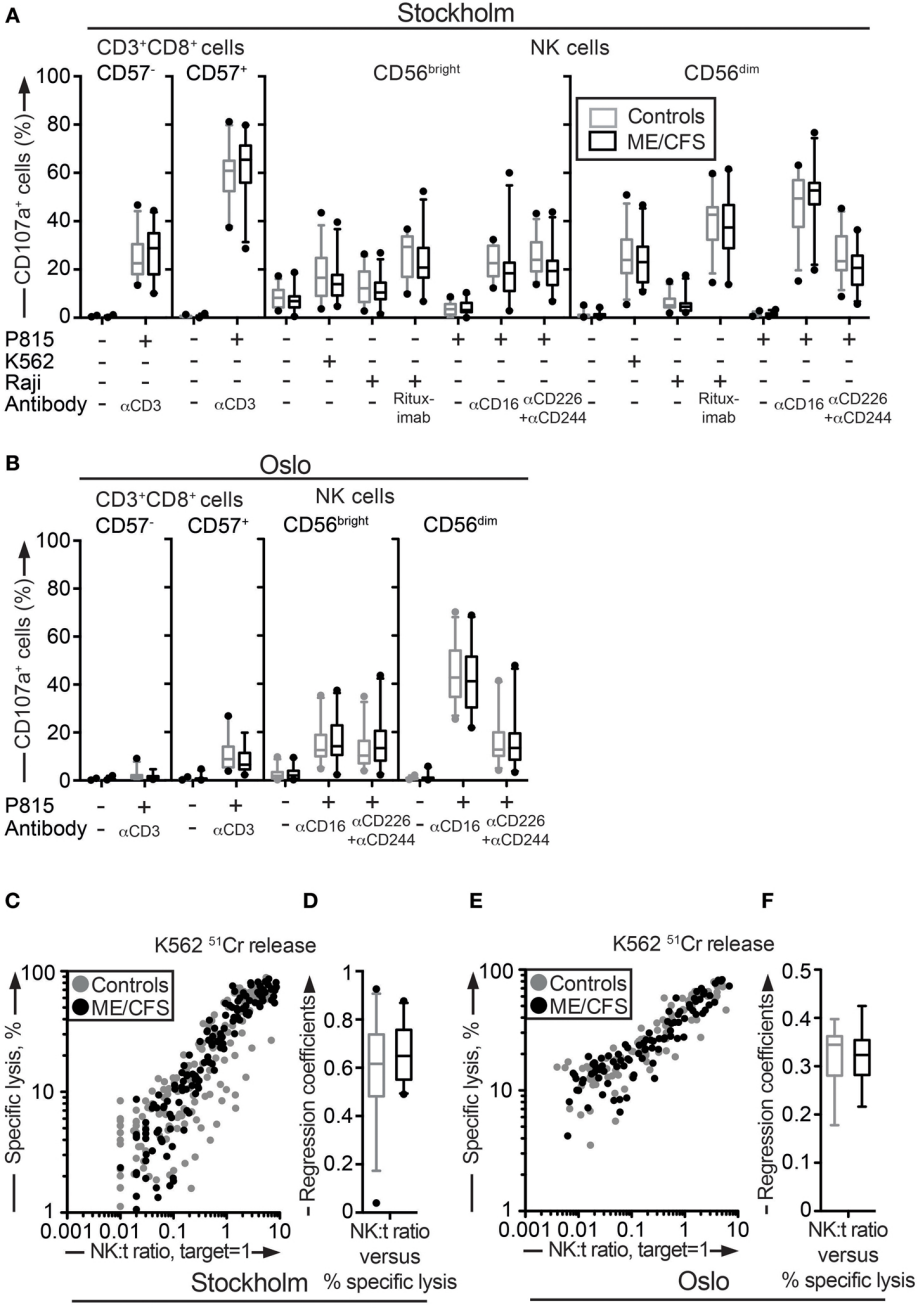
Assessment of exocytosis as well as K562 killing are not different between ME/CFS patients and controls. **(A,B)** Analysis of exocytosis. PBMC were thawed, rested overnight and then stimulated for 4 h with no target, P815 + anti-CD3, K562, Raji, Raji + Rituximab, P815, P815 + anti-CD16, P815 + anti-CD244 (2B4) + antiCD226 (DNAM-1), and subsequently stained with antibodies to CD107a in combination with lineage markers. Percentage of four CD8^+^ T cell and NK cell subsets positive for CD107a in the Stockholm and Oslo substudy respectively is shown. Gray boxes depict control values, whereas black boxes depict patient values. Lines through boxes show the median. Error bars extend to 5th and 95th percentile. Dots show outliers. **(C,E)** K562 lysis of NK cells from the Stockholm and Oslo substudies. NK cell activity measured with K562 lysis. K562 cells were labeled with ^51^Cr, and subsequently co-cultured with PBMC for 4 h at six standardized PBMC concentrations. Subsequently, gamma-emissions from the supernatants were registered. *X*-axis shows the NK:target ratio, with the target set to 1. The NK cell values are estimated from average percentage of NK cells in simultaneously performed flow cytometric assay. *Y*-axis shows specific lysis in percent, calculated with the formula (100 × (*x* − min)/(max − min)), where *x* = average value from duplicates or triplicates (Stockholm and Oslo analysis, respectively), min = average value from negative control triplets, max = average value from positive control triplets. Gray dots depict control values; black dots depict patient values. **(D,F)** Regression coefficients for the specific lysis in percent as a function of the NK:target ratio calculated for every individual for the Stockholm and Oslo analysis, respectively. Gray boxes depict control values, whereas black boxes depict patient values. Lines through boxes show the median. Error bars extend to 5th and 95th percentile. Dots show outliers. 24 patients and 28 controls for the Stockholm substudy and, 22 patients and 24 controls [15 patients and 16 controls for **(E,F)**] for the Oslo substudy, are included in the analyses.

Dysfunction in lymphocyte cytotoxicity has been denoted a hallmark of ME/CFS (42). Apart from perforin deficiency, the majority of the molecularly defined defects in lymphocyte cytotoxicity arise from aberrances cytotoxic granule exocytosis (19). Parallel valuation of T cell and NK cell responses can increase sensitivity for the identification of primary defects in cytotoxic granule exocytosis (23). Furthermore, NK cells can be activated through multiple co-activating receptor combinations (43). Thus, potential selective defects in specific receptor signaling pathways may only be identified through comprehensive screening of different stimulations. For these reasons, the exocytic capacity of specific CD8^+^ T cell and NK cell subsets from ME/CFS patients was investigated following multiple different stimuli. PBMC were thawed and rested overnight in complete medium. The cells were subsequently stimulated with medium alone, P815 cells, P815 cells supplemented with anti-CD3 antibody (clone S4.1), P815 cells supplemented with anti-CD16 or with anti-CD226 (DNAM-1) and anti-CD244 (2B4) antibodies, K562 cells, Raji cells, Raji cells supplemented with Rituximab (inducing NK cell antibody-dependent cellular cytotoxicity). After 4 h, the cells were surface stained with antibodies to lineage markers, as well as CD107a, and analyzed by flow cytometry. No significant differences were seen between the patients and the controls for any of the subsets or stimulations (Figure 2A). Moreover, in a follow-up experiment in the Oslo substudy with a similar setup, but where only P815, P815 with anti-CD3 (clone UCHT.1), P815 cells supplemented with anti-CD16 or with anti-CD226 (DNAM-1), and anti-CD244 (2B4) antibodies were investigated, no differences were observed (Figure 2B). In addition, there was no difference in regards to the number of low responders between the controls and the patients (Figures 2A,B).

Deficient NK cell-mediated cytotoxicity has previously been linked to ME/CFS (12, 44). Although cytotoxic lymphocyte granule content and exocytic capacity was not impaired in the ME/CFS patients, we nonetheless examined the ability of NK cells to kill target cells using a ^51^Cr release assay. ^51^Cr-labeled K562 target cells are frequently employed for the diagnostics of patients with defective lymphocyte cytotoxicity (45). PBMC from all patients and controls in the Stockholm substudy were simultaneously thawed, rested overnight in complete medium, and evaluated for lysis of ^51^Cr-labeled K562 cells. Killing by NK cells from ME/CFS and healthy controls was similar at the examined effector to target ratios (Figure 2C). Following this, an analysis of the linear regression coefficients for the specific lysis in percent as a function of the NK cell percentage among PBMC was performed for each individual, reflecting the per-cell cytotoxic efficiency. Comparison between the patient and healthy control group revealed no significant difference (Figure 2D). A few outliers with a lower cytotoxicity were identified, but these were all among the healthy controls (Figures 2C,D). An identical experiment with 15 and 16 of the Oslo patients and controls, respectively, was also performed. Consistent with results from the Stockholm substudy, no differences between the groups were seen (Figures 2E,F). Thus, neither the release of cytotoxic granules after multiple different stimuli nor cytotoxicity was impaired in ME/CFS patients as compared to controls.

### Evaluation of Peripheral Blood Cytotoxic Lymphocyte Pro-inflammatory Cytokine Production

**Figure 3:**
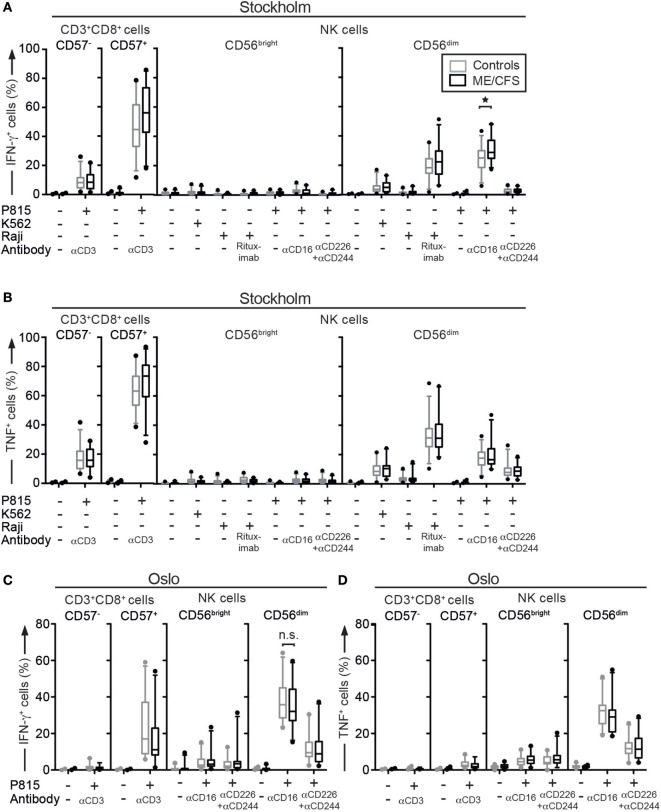
Pro-inflammatory cytokine production is similar between ME/CFS patients and controls. **(A–D)** Analysis of cytokine production. Peripheral blood mononuclear cells were thawed, rested overnight, and then stimulated for 4 h with no target, P815 + anti-CD3, K562, Raji, Raji + Rituximab, P815, P815 + anti-CD16, P815 + anti-CD244 + anti-CD226 (DNAM-1), and subsequently stained with antibodies to lineage markers, fixed, permeabilized, and stained intracellularly for IFN-γ and TNF. **(A)** Percentage of four CD8^+^ T cell and natural killer (NK) cell subsets positive for IFN-γ in the Stockholm substudy. **(B)** Percentage of four CD8^+^ T cell and NK cell subsets positive for TNF in the Stockholm substudy. **(C)** Percentage of four CD8^+^ T cell and NK cell subsets positive for IFN-γ in the Oslo substudy. **(D)** Percentage of four CD8^+^ T cell and NK cell subsets positive for TNF in the Oslo substudy. Gray boxes depict control values, whereas black boxes depict patient values. Lines through boxes show the median. Error bars extend to 5th and 95th percentile. Dots show outliers. 24 patients and 28 controls and 22 patients and 24 controls for the Stockholm and Oslo substudies, respectively, are included in the analyses.

In addition to killing, target cell-mediated activation of cytotoxic lymphocyte induces production of pro-inflammatory cytokines. Such cytokines are central to the defense against intracellular pathogens. To evaluate cytokine production ability, PBMC from the Stockholm substudy were thawed and rested overnight in complete medium. The cells were subsequently stimulated with medium alone, P815 cells, P815 cells supplemented with anti-CD3 antibody (clone S4.1), P815 cells supplemented with anti-CD16 or with anti-CD226 (DNAM-1) and anti-CD244 (2B4) antibodies, K562 cells, Raji cells, and Raji cells supplemented with Rituximab. After 4 h, the cells were surface stained with antibodies to lineage markers, fixed, permeabilized, stained intracellularly with antibodies to IFN-γ and TNF, and analyzed by flow cytometry. CD56^dim^ NK cells from ME/CFS patients produced more IFN-γ after stimulation with P815 and anti-CD16 antibody than controls (*p* = 0.009, not significant after correction for multiple testing), and a similar trend was seen for CD56^bright^ NK cells (Figure 3A). No clear differences were noted for TNF (Figure 3B). A follow-up experiment was performed for the Oslo substudy with a similar setup, but where only P815, P815 with anti-CD3 (clone UCHT.1), and P815 cells supplemented with anti-CD16 or with anti-CD226 and anti-CD244 antibodies were investigated. Here, no differences between the patients and controls were observed (Figures 3C,D). Thus, although a trend toward cytokine hyperresponsivity by ME/CFS patients was noted in the Stockholm substudy, no such trend was observed in the Oslo substudy.

### Assessment of Adaptive NK Cell Frequencies in ME/CFS Patients

**Figure 4:**
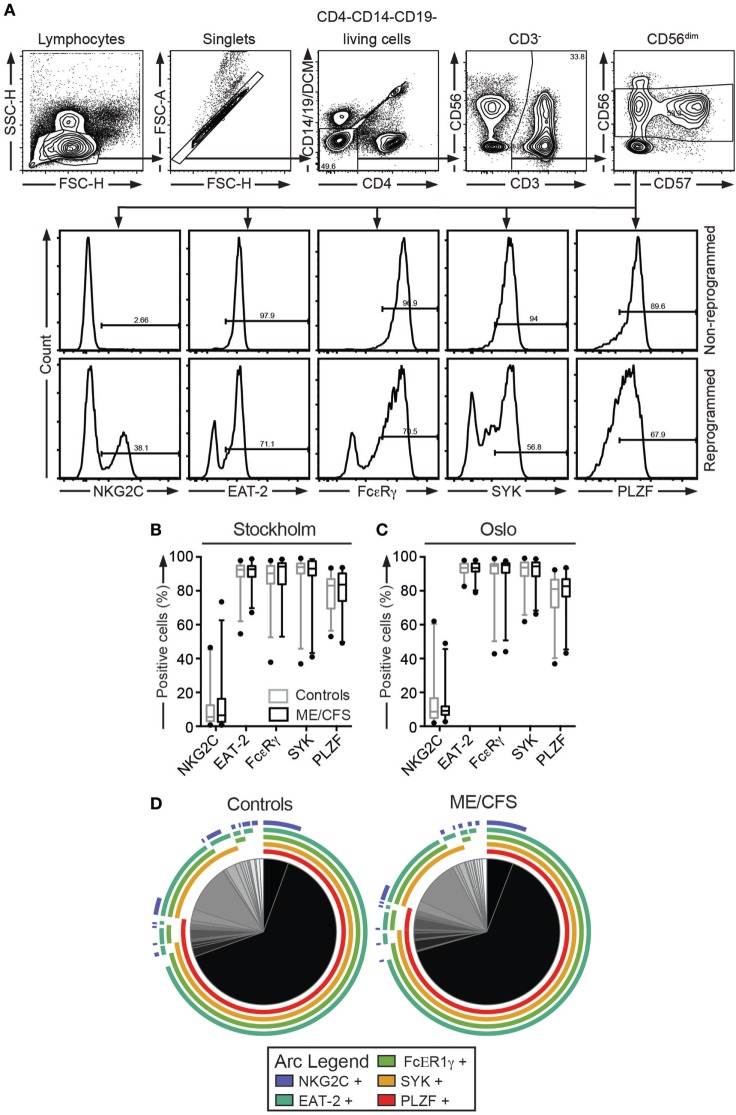
Investigation of expanded adaptive natural killer (NK) cell subsets does not reveal any differences between ME/CFS patients and controls. **(A–D)** Analysis of percentages of adaptive NK cell expansions. Peripheral blood mononuclear cells were thawed and stained with antibodies to lineage markers, NKG2C, and a dead cell marker, fixed, permeabilized, and stained intracellularly for FcεRγ, SYK, EAT-2, and PLZF. **(A)** Gating strategy for one representative donor is shown. Histograms depict one donor without and one donor with adaptive NK cell expansions. **(B,C)** Percentage of CD3^−^CD56^dim^ NK cells positive for NKG2C, FcεRγ, SYK, EAT-2, and PLZF in the Stockholm and Oslo substudies, respectively. Adaptive NK cells express combinations of high NKG2C and low FcεRγ, SYK, EAT-2, and PLZF. Gray boxes depict control values, whereas black boxes depict patient values. Lines through boxes show the median. Error bars extend to 5th and 95th percentile. Dots show outliers. **(D)** Pie charts of Boolean analyses of subsets expressing any combination of NKG2C, FcεRγ, SYK, EAT-2, and PLZF. If an arc is drawn over a specific pie slice, then the connected marker is present on the pie slice in question. Following this, the majority of cells are positive for FcεRγ, SYK, EAT-2, and PLZF, and thus non-adaptive. 24 patients and 28 controls and 24 patients and 24 controls for the Stockholm and Oslo substudies, respectively, are included in the analyses.

After the investigation of direct aberrances in phenotype and function of cytotoxic lymphocytes in ME/CFS, evaluation cytotoxic lymphocyte functionality resulting from environmental exposures followed. As a first step, the frequencies of adaptive CD56^dim^ NK cell subsets from the Stockholm and Oslo substudies were determined, as this cell population displays reduced immunoregulatory capacity and is strongly associated with CMV infection. PBMC were thawed, surface stained with antibodies to lineage markers and NKG2C, fixed, permeabilized, and stained intracellularly with antibodies to FcεRγ EAT-2, SYK, and PLZF. Increased frequencies of adaptive CD56^dim^ NK cell subsets were not observed in samples from either Stockholm or Oslo (Figures 4A–C). To investigate the coexpression patterns of NKG2C, FcεRγ EAT-2, SYK, and PLZF in NK cells, a Boolean gating strategy was applied that identified all possible combinations of these markers in the Oslo substudy. There was no subset with any combination of markers that differed between the patient and control groups (Figure 4D). Thus, the hypothesis that expansions of epigenetically modified NK cells may play a role in ME/CFS is not supported.

### Adrenergic Regulation of Peripheral Blood Cytotoxic Lymphocyte Function

**Figure 5:**
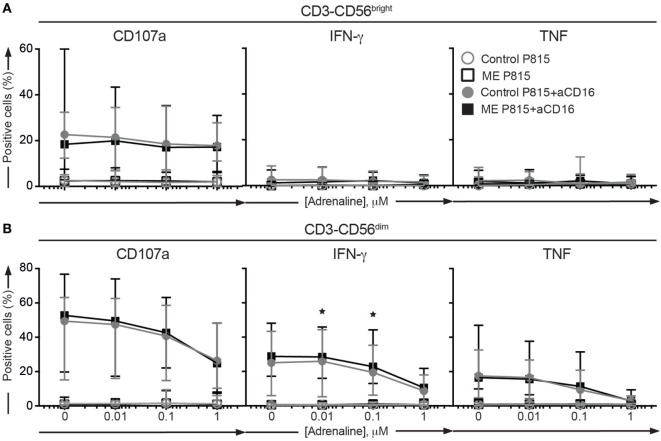
Adrenaline inhibition is not significantly different between ME/CFS patients and controls. **(A,B)** Analysis of exocytosis, IFN-γ, and TNF production after inhibition with adrenaline in the Stockholm substudy. Peripheral blood mononuclear cells were thawed, rested overnight, preincubated for 30 min without or with adrenaline at three concentrations and then stimulated for 4 h with P815 or P815 + anti-CD16, and subsequently stained with antibodies to CD107a in combination with lineage markers, fixed, permeabilized, and stained intracellularly for IFN-γ and TNF. **(A)** Percentage of CD3^−^CD56^bright^ natural killer (NK) cells positive for CD107a, IFN-γ, or TNF. **(B)** Percentage of CD3^−^CD56^dim^ NK cells positive for CD107a, IFN-γ, or TNF. 24 patients and 28 controls from the Stockholm substudy are included in the analyses.

Physical and psychological stress can precipitate ME/CFS symptoms (2). Cytotoxic lymphocytes function is attenuated by adrenaline-mediated stress responses, thus providing a possible link between ME/CFS and impairments in cytotoxic lymphocytes. To probe for a possible aberrance in the inhibitory capacity of adrenaline on cytotoxic lymphocyte function in ME/CFS, an experiment in the Stockholm substudy was performed. PBMC were thawed, rested overnight in complete medium, and subsequently preincubated with 1, 0.1, and 0.01 µM of adrenaline or medium alone. Thereafter, P815 cells or P815 cells supplemented with anti-CD16 antibody were added to each well. After 4 h, the cells were stained with antibodies to lineage markers and CD107a, fixed, permeabilized, and stained intracellularly with antibodies to IFN-γ and TNF (Figures 5A,B). The CD56^dim^ cells from ME/CFS patients tended to display less inhibition of the IFN-γ production at 0.1 and 0.01 µM compared to the controls (*p* = 0.004 and 0.009, respectively, significant after correction for multiple comparisons) (Figure 5B). In summary, a slightly weaker effect of adrenaline inhibition on cytokine production from cytotoxic lymphocytes after receptor stimuli could be present.

### Identification of Patient/Control Discriminators in the Full, Multidimensional Dataset

Individual analyses of cytotoxic lymphocyte phenotypic or functional parameters might not identify patterns of mild deficiencies that taken together could have an impact on lymphocyte cytotoxicity. Moreover, studies have indicated that subgroups of ME/CFS patients may exist (46–48). Thus, to identify such potential subgroups and patterns of mild deficiencies, supervised and unsupervised dimensionality reduction techniques were utilized on the full substudy datasets.

**Figure 6:**
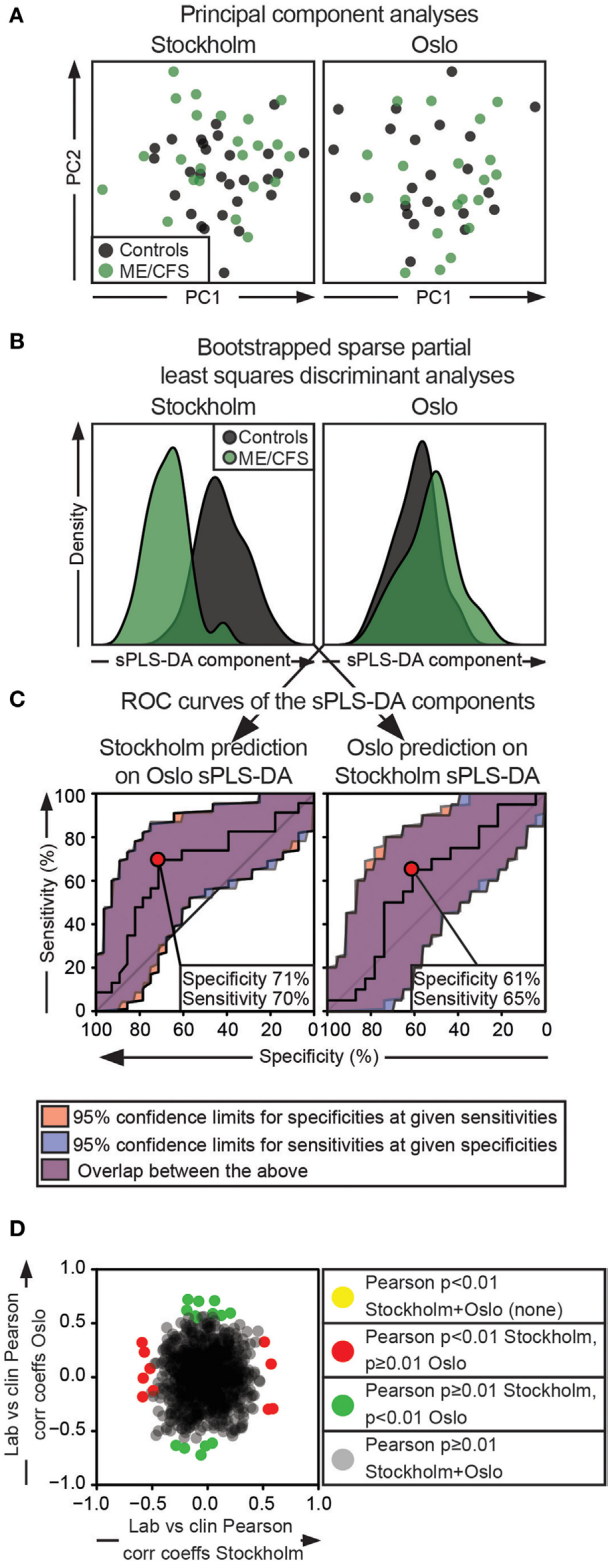
Multidimensionality approaches do not reveal parameter combinations that can separate both Oslo and Stockholm patients from controls. Separate analyses for the Stockholm and Oslo substudies are represented in the first and second column, respectively. **(A)** Principal component (PC) analysis. *X*- and *Y*-axes show the estimated PC1 and PC2, respectively. Black and green dots indicate control and ME/CFS individuals, respectively. **(B)** Histograms show the sparse partial least squares discriminant components for the separate analyses. Gray and green colors indicate controls and ME/CFS patients, respectively. **(C)** Receiver-operating characteristic (ROC) curves are shown. The left ROC curve is based on a crossover discriminant component of Stockholm dataset generated by multiplying the variable and the loading identified in the Oslo sPLS-DA. The right ROC curve is generated in the corresponding way for the Oslo dataset. **(D)** Oslo Pearson product-moment correlation coefficients are plotted as a function of Stockholm Pearson product-moment correlation coefficients for correlations of clinical vs cytotoxic lymphocyte parameters. Gray dots show correlations that are insignificant in both substudies. Red and green dots show significant correlations in the Stockholm and Oslo substudies, respectively. Yellow dots should show correlations that overlap between the analyses (none present). For the Stockholm and Oslo substudies, respectively, 23 + 28 and 20 + 23 patients + controls are included in the analyses.

To elucidate whether the participant class (being a patient or control) was contributing to the overall variance in the dataset, PC analyses including all 51 common investigated parameters (Table 2), were conducted for all the complete cases from the Stockholm and Oslo substudies separately. Briefly, the first PC is defined as the vector through a multidimensional data cloud that explains the most variance. The second PC is then defined as the vector with the second most variance, with the constraint that this vector must be orthogonal to the first PC. All following PCs relate to the previous ones in the same way. In this unsupervised method, the first 14 PCs were investigated. These explained 81 and 86% of the total variance in the Stockholm and Oslo data, respectively (Figure S2A and Table S3 in Supplementary Material). In the Stockholm analysis, patient/control separation was evident in the fifth PC. This PC explained 7% of the Stockholm dataset variance. Thus, it could be assumed that participant class explained ≈7% of the variance in the Stockholm dataset. No class separation was evident in the Oslo PCA. In addition, no clusters of patient subgroups were evident in any of the components (Figure 6A; Figure S2B in Supplementary Material).

**Table 2:** Parameters common to the Stockholm and Oslo substudies used for identification of patient/control discriminators.

		CD3^+^CD4^−^CD8^+^		CD3^−^	
		CD57^−^	CD57^+^	CD56^bright^	CD56^dim^
Perforin		x	x	x	x
Granzyme A		x	x	x	x
Granzyme B		x	x	x	x
CD38		x	x	x	x
CD69		x	x	x	x
CD279		x	x	x	x
HLA-DR		x	x	x	x
P815+anti-CD3: CD107a		x	x	–	–
P815+anti-CD3: IFN-γ		x	x	–	–
P815+anti-CD3: TNF		x	x	–	–
P815+anti-CD16: CD107a		–	–	x	x
P815+anti-CD16: IFN-γ		–	–	x	x
P815+anti-CD16: TNF		–	–	x	x
P815+anti-CD226+anti-CD244: CD107a		–	–	x	x
P815+anti-CD226+anti-CD244: IFN-γ		–	–	x	x
P815+anti-CD226+anti-CD244: TNF		–	–	x	x
NKG2C		–	–	–	x
EAT-2		–	–	–	x
FcεRγ		–	–	–	x
SYK		–	–	–	x
PLZF		–	–	–	x
x	Included parameter		Parameter contributing to Stockholm sPLS-DA vector		
–	Not included parameter				
			Parameter contributing to Oslo sPLS-DA vector		
			Parameter contributing to both sPLS-DA (none)		

To explore the separation of patients and controls in the Stockholm substudy further and to search for any meaningful separation of the groups in the Oslo substudy, sparse projection to latent structures discriminant analyses (sPLS-DA) were performed on all 51 common, transformed immunological parameters for the Stockholm and the Oslo substudies separately. sPLS-DA is a supervised method that identifies the vector(s) through a multidimensional data cloud that separates the predefined classes at maximum. By using a penalty for low or absent contribution, variables that contribute to such a discriminant vector are identified (49). As the dataset in this case was structured into two classes (patient or control), models with one discriminant vector were chosen in accordance with previous literature (50). To increase the robustness of the analysis, a bootstrapped sPLS-DA algorithm was used (51). For the Stockholm and Oslo substudy analysis, respectively, 14 and 1 immunological parameters were identified that contributed to the discriminant vector (Table 2; Figure 6B). Following this, the discriminant vector from Stockholm was reconstructed by multiplying the same immunological parameters from Oslo, with the Stockholm discriminant vector loadings, and *vice versa*. These “synthetic” discriminant vectors were then used for prediction purposes by generating receiver-operating characteristic curves (Figure 6C). In this analysis, the optimal prediction for the Stockholm substudy based on the result from the Oslo sPLS-DA was around 70% in specificity and sensitivity. In the opposite analysis, the optimal sensitivity/specificity was around 60%. Thus, none of the identified discriminant vectors could separate cases from controls in the other substudy.

Possible subgrouping based on clinical parameters was explored. Patients included in this study had generally been suffering of ME/CFS for >2 years, and an infectious trigger was present in a clear majority of the cases, preventing subgrouping in these parameters. However, 20 clinical assessments of pain, fatigue, muscle pain, etc. (listed in Table S1 in Supplementary Material) were available for all patients. Hypothetically, identifying clinical parameters that correlate to laboratory findings could provide a base for further stratification of subgroups. Thus, Pearson product-moment correlations between the 51 cytotoxic lymphocyte parameters shown in Table 2, and the 21 clinical assessments were performed for the Stockholm and Oslo substudies individually. This generated two matrices with 21 × 51 correlation coefficients and two equally sized matrices with the correlation *p*-values. When these 1,071 correlation coefficients and their respective *p*-values were compared, no correlation was less than 0.01 for both substudies (Figure 6D). No further subgroup stratification was therefore attempted. In summary, no patterns of laboratory or clinical findings separated all or subgroups of patients from the controls either using unsupervised or supervised methods.

## Discussion

Traditionally, primary immunodeficiencies (PIDs) have been regarded as severe monogenetic defects with high penetrance, resulting in often fatal syndromes in infants. This view is changing, and PIDs are rather viewed as a spectrum of disorders ranging from fatal syndromes in infancy to diseases presenting in adulthood, limited to recurrent infections with specific pathogens or even including autoimmunity (52). Whereas autosomal recessive mutations abrogating cytotoxic function invariably cause early-onset familial hemophagocytic lymphohistiocytosis, less severe impairments have been associated with a spectrum of disorders ranging from hematologic malignancies to neurologic manifestations (19). Heterozygous carriership of such mutations increases the risk of malignancy (20). Notably, animal models and certain observations in humans implicate intracellular infections as triggers of diverse clinical manifestations. Prompted by an association between intracellular infections and development of the ME/CFS as well as several reports of impaired NK cell function in ME/CFS patients, we investigated if ME/CFS might represent a relatively mild manifestation of PID caused by impaired lymphocyte cytotoxicity. To this end, recently improved, sensitive and specific assays that readily identify patients with primary defects in lymphocyte cytotoxicity were used (23, 53). Our analyses did not uncover any systematic evidence for defects in lymphocyte cytotoxicity, in individual patients or the patient groups as a whole. One individual was homozygous, and four were heterozygous for the *PRF1* p.A91V variant, associated with impaired lymphocyte cytotoxicity and an increased risk of certain malignancies (54). However, *PRF1* p.A91V homozygous individuals are reported in studies of healthy populations and the risk for disease development in such individuals remains unclear (55). In our limited material, the overall frequency of this variant was not significantly increased among our ME/CFS patients.

Latent CMV infection affects numerous immunological parameters (56). In many individuals, an effect of CMV infection is the expansion of epigenetically altered adaptive NK cells with limited immunoregulatory functions (24, 57). Hypothetically, a genetic or epigenetic predisposition in combination with CMV infection could generate an immune system with limited immunoregulatory capacities. This could explain how common infections might cause unremitting inflammation in ME/CFS patients, sparking our investigation of adaptive NK cells. We found no indications of any differences between ME/CFS patients and controls. To the best of our knowledge, this is the first report to study the prevalence of adaptive NK cells in the context of ME/CFS.

A central feature of ME/CFS is post-exertional exhaustion (4, 58). Notably, β2-adrenergic stimulation can directly inhibit CTL and NK cell functions (29, 31, 59, 60). Hypothalamic–neuroendocrine–adrenal axis dysregulation and other neuroendocrine disturbances are features of ME/CFS (18). When probing the response inhibition by adrenaline in the Stockholm substudy, IFN-γ expression by CD56^dim^ NK cells was less inhibited by adrenaline in cells from ME/CFS patients. This is in line with a previous study, showing lower effect of the β2-adrenergic receptor agonist terbutaline on inhibiting monocyte TNF production (61). Our potentially confirmatory experiment on the Oslo cohort regrettably failed for technical reasons.

In many autoimmune disorders, interactions between multiple predisposing factors and environmental triggers cause disease. In an attempt to investigate if this could be the case for cytotoxic lymphocyte dysfunction and ME/CFS, we deployed both unsupervised and supervised methods considering all investigated cytotoxic lymphocyte functional and phenotypic parameters together. There was no tendency toward any such reproducible difference. Furthermore, identifying subgroups among the patients based on both clinical observations and laboratory findings was not possible. These findings indicate that none of the parameters studied here are useful in a clinical setting in differentiating ME/CFS patients from other individuals. Further strengthening this notion, even by increasing the accepted α-error rate to 5%, no single parameter was significant for both the Stockholm and the Oslo substudies (Figure S3 in Supplementary Material).

It is worth noting that a number of differences were identified in the Stockholm substudy, whereas no differences were noted in the Oslo substudy. A likely explanation for this is that the Stockholm substudy sample collection, PBMC isolation, and freezing protocols slightly differed between patients and controls, whereas the Oslo patient and control samples were handled identically in all steps. An arguable difference between the substudies lies in the average age that differed 14 years between the Stockholm and the Oslo substudies. Our previous work has not shown any correlation between age and cytotoxic lymphocyte function (23) nor frequency of adaptive NK cells (24) in healthy individuals. Moreover, of the common parameters to these substudies exhibiting a *p*-value lower than 0.1, the highest Pearson product-moment correlation coefficient to age was 0.3. Hence, the age difference between the substudies is unlikely to influence the results. In terms of symptoms, patients from Oslo showed higher levels of concentration problems than the Stockholm patients (Table S1 in Supplementary Material, *p* = 0.001, significant after correction for multiple comparisons), but this was the only significant difference between the patient populations. Thus, it is unlikely that the discrepancy in the number of differences between the substudies can be attributed to differences in the patient populations.

Our negative results contrast a number of previously published studies indicating diminished NK cell function in ME/CFS (for a comprehensive list of relevant publications overall content and outcome, see Table S4 in Supplementary Material). A few studies have shown that K562 cell killing was diminished in whole-blood assays (13, 37). Other studies found similarly diminished NK cell cytotoxic activity using PBMC immediately after isolation (15, 46), whereas other investigators have not found any impairments of NK cell cytotoxic activity using isolated PBMC (16, 62). Timing of functional assays *vis-à-vis* PBMC isolation may explain differences, with an effect of soluble factors, such as cytokines, catecholamines, and hormones, waning with time from cell isolation from whole blood. Such a discrepancy would therefore suggest that NK cells from ME/CFS patients in general are intrinsically normal but might be responding to an abnormal external milieu, rendering them hypofunctional in whole blood or immediately after isolation. Congruently, the addition of adrenaline, as shown above, did not attenuate NK cell functionality more in the patients than in the controls. If anything, the opposite was observed, which could hypothetically be explained by the presence of β2-adrenergic receptor autoantibodies (63), or downregulations of β2-adrenergic receptors due to an increased tonic adrenergic signaling in the ME/CFS patients. Regardless, our results indicate that cell intrinsic differences in cytotoxic lymphocyte differentiation or function do not clearly distinguish ME/CFS patients from controls.

In conclusion, none of the evaluated aspects of cytotoxic lymphocyte phenotype or function systematically separated ME/CFS patients from controls. As such, our investigation does not indicate impairments in cytotoxic lymphocyte differentiation or function as a major cause of ME/CFS. Thus, these results do not support the use of these cytotoxic lymphocyte phenotypic or functional examinations for biomarker purposes in clinical diagnostic procedures of ME/CFS.

## Ethics Statement

The study was approved by regional Ethical Review Boards in Stockholm (EPN Stockholm) and Oslo (REK Sør-Øst C). Written informed consent was obtained from all patients as well as from the healthy controls recruited from the Oslo University Hospital Blood Bank. Oral informed consent was obtained from healthy controls recruited from the Stockholm Blood Bank.

## Author Contributions

JT collected patient samples, designed and performed experiments, analyzed data, and wrote the manuscript; IB-L and BA-A recruited patients, collected patient samples and clinical data, and contributed to drafting the manuscript; BT designed, performed, and analyzed genotyping experiments and contributed to drafting the manuscript; HS developed flow cytometry assays and contributed to drafting the manuscript; MJ provided pilot study material and contributed to drafting the manuscript; ES recruited patients and contributed to drafting the manuscript; YB coordinated research efforts, supervised research and data analysis, and wrote the manuscript; and all authors discussed and revised the manuscript.

## Conflict of Interest Statement

The authors declare that the research was conducted in the absence of any commercial or financial relationships that could be construed as a potential conflict of interest.
