# Corrigendum: Unperturbed Cytotoxic Lymphocyte Phenotype and Function in Myalgic Encephalomyelitis/Chronic Fatigue Syndrome Patients

**DOI:** 10.3389/fimmu.2019.00350

**Published:** 2019-03-11

**Authors:** Jakob Theorell, Indre Bileviciute-Ljungar, Bianca Tesi, Heinrich Schlums, Mette Sophie Johnsgaard, Babak Asadi-Azarbaijani, Elin Bolle Strand, Yenan T. Bryceson

**Affiliations:** ^1^Department of Medicine, Huddinge, Karolinska Institutet, Stockholm, Sweden; ^2^Department of Rehabilitation Medicine, Karolinska Institutet, Stockholm, Sweden; ^3^Department of Clinical Sciences, Danderyd Hospital, Karolinska Institutet, Stockholm, Sweden; ^4^Childhood Cancer Research Unit, Department of Women's and Children's Health, Karolinska Institute, Karolinska University Hospital Solna, Stockholm, Sweden; ^5^Clinical Genetics Unit, Department of Molecular Medicine and Surgery, Center for Molecular Medicine, Karolinska Institute, Karolinska University Hospital Solna, Stockholm, Sweden; ^6^Balderklinikken, Oslo, Norway; ^7^Division of Medicine, CFS/ME Centre, Oslo University Hospital, Oslo, Norway; ^8^VID Specialized University, Oslo, Norway; ^9^Norwegian National Advisory Unit on CFS/ME, Oslo University Hospital, Oslo, Norway; ^10^Department of Clinical Science, University of Bergen, Bergen, Norway

**Keywords:** chronic fatigue syndrome, myalgic encephalomyelitis, natural killer cells, cytotoxic T cells, lymphocyte cytotoxicity, adaptive natural killer cells

In the original article, there was a consistent misuse of the term “lytic units”. In all places where it occurred, it has now been replaced with “specific lysis in percent”. The following sections detail where the errors occurred.

There was a mistake in the legend for Figures 2D,F as published. The term “lytic units” was used incorrectly. It should be replaced by “specific lysis in percent.” The correct legend appears below.

**Figure 2:**
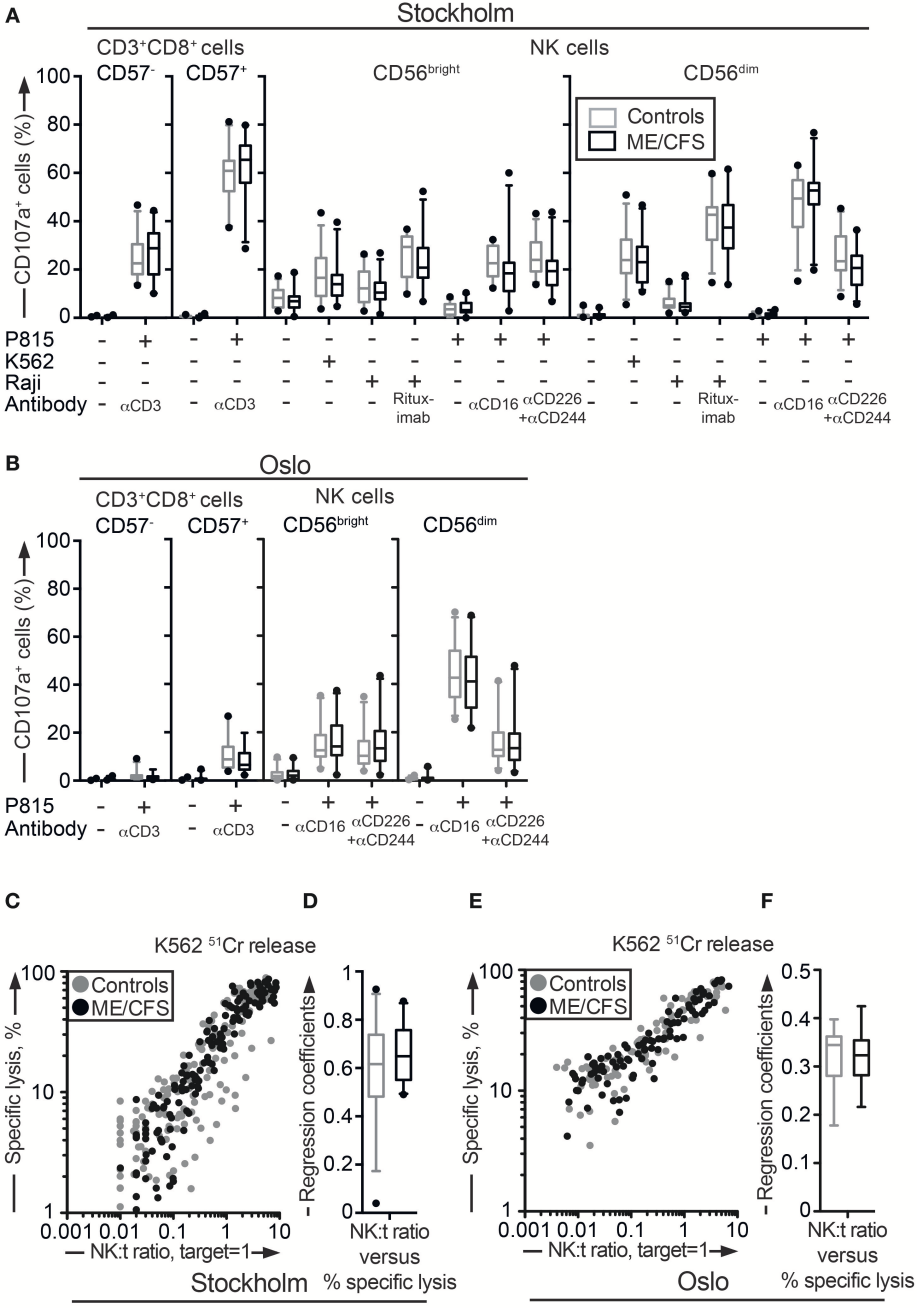
Assessment of exocytosis as well as K562 killing are not different between ME/CFS patients and controls. **(A,B)** Analysis of exocytosis. PBMC were thawed, rested overnight and then stimulated for 4 h with no target, P815 + anti-CD3, K562, Raji, Raji + Rituximab, P815, P815 + anti-CD16, P815 + anti-CD244 (2B4) + antiCD226 (DNAM-1), and subsequently stained with antibodies to CD107a in combination with lineage markers. Percentage of four CD8^+^ T cell and NK cell subsets positive for CD107a in the Stockholm and Oslo substudy respectively is shown. Gray boxes depict control values, whereas black boxes depict patient values. Lines through boxes show the median. Error bars extend to 5th and 95th percentile. Dots show outliers. **(C,E)** K562 lysis of NK cells from the Stockholm and Oslo substudies. NK cell activity measured with K562 lysis. K562 cells were labeled with ^51^Cr, and subsequently co-cultured with PBMC for 4 h at six standardized PBMC concentrations. Subsequently, gamma-emissions from the supernatants were registered. *X*-axis shows the NK:target ratio, with the target set to 1. The NK cell values are estimated from average percentage of NK cells in simultaneously performed flow cytometric assay. *Y*-axis shows specific lysis in percent, calculated with the formula (100 × (*x* − min)/(max − min)), where *x* = average value from duplicates or triplicates (Stockholm and Oslo analysis, respectively), min = average value from negative control triplets, max = average value from positive control triplets. Gray dots depict control values; black dots depict patient values. **(D,F)** Regression coefficients for the specific lysis in percent as a function of the NK:target ratio calculated for every individual for the Stockholm and Oslo analysis, respectively. Gray boxes depict control values, whereas black boxes depict patient values. Lines through boxes show the median. Error bars extend to 5th and 95th percentile. Dots show outliers. 24 patients and 28 controls for the Stockholm substudy and, 22 patients and 24 controls [15 patients and 16 controls for **(E,F)**] for the Oslo substudy, are included in the analyses.

“Assessment of exocytosis as well as K562 killing are not different between ME/CFS patients and controls. **(A,B)** Analysis of exocytosis. PBMC were thawed, rested overnight and then stimulated for 4 h with no target, P815 + anti-CD3, K562, Raji, Raji + Rituximab, P815, P815 + anti-CD16, P815 + anti-CD244 (2B4) + antiCD226 (DNAM-1), and subsequently stained with antibodies to CD107a in combination with lineage markers. Percentage of four CD8^+^ T cell and NK cell subsets positive for CD107a in the Stockholm and Oslo substudy respectively is shown. Gray boxes depict control values, whereas black boxes depict patient values. Lines through boxes show the median. Error bars extend to 5th and 95th percentile. Dots show outliers. **(C,E)** K562 lysis of NK cells from the Stockholm and Oslo substudies. NK cell activity measured with K562 lysis. K562 cells were labeled with ^51^Cr, and subsequently co-cultured with PBMC for 4 h at six standardized PBMC concentrations. Subsequently, gamma-emissions from the supernatants were registered. *X*-axis shows the NK:target ratio, with the target set to 1. The NK cell values are estimated from average percentage of NK cells in simultaneously performed flow cytometric assay. *Y*-axis shows specific lysis in percent, calculated with the formula (100 × (*x* − min)/(max − min)), where *x* = average value from duplicates or triplicates (Stockholm and Oslo analysis, respectively), min = average value from negative control triplets, max = average value from positive control triplets. Gray dots depict control values; black dots depict patient values. **(D,F)** Regression coefficients for the specific lysis in percent as a function of the NK:target ratio calculated for every individual for the Stockholm and Oslo analysis, respectively. Gray boxes depict control values, whereas black boxes depict patient values. Lines through boxes show the median. Error bars extend to 5th and 95th percentile. Dots show outliers. 24 patients and 28 controls for the Stockholm substudy and, 22 patients and 24 controls [15 patients and 16 controls for **(E,F)**] for the Oslo substudy, are included in the analyses.”

Additionally, there was a mistake in Figure 2 as published. The term “lytic units” was used incorrectly. It should be replaced by “Specific lysis, %.” The corrected Figure 2 appears above.

Similarly, in the original article, there was an error. The term “lytic units” is used incorrectly. It should be replaced by “specific lysis in percent”. A correction has been made to the **Results**, **Evaluation of peripheral blood cytotoxic lymphocyte function**, **Paragraph two**:

“Deficient NK cell-mediated cytotoxicity has previously been linked to ME/CFS (12, 44). Although cytotoxic lymphocyte granule content and exocytic capacity was not impaired in the ME/CFS patients, we nonetheless examined the ability of NK cells to kill target cells using a ^51^Cr release assay. ^51^Cr-labeled K562 target cells are frequently employed for the diagnostics of patients with defective lymphocyte cytotoxicity (45). PBMC from all patients and controls in the Stockholm substudy were simultaneously thawed, rested overnight in complete medium, and evaluated for lysis of ^51^Cr-labeled K562 cells. Killing by NK cells from ME/CFS and healthy controls was similar at the examined effector to target ratios (Figure 2C). Following this, an analysis of the linear regression coefficients for the specific lysis in percent as a function of the NK cell percentage among PBMC was performed for each individual, reflecting the per-cell cytotoxic efficiency. Comparison between the patient and healthy control group revealed no significant difference (Figure 2D). A few outliers with a lower cytotoxicity were identified, but these were all among the healthy controls (Figures 2C,D). An identical experiment with 15 and 16 of the Oslo patients and controls, respectively, was also performed. Consistent with results from the Stockholm substudy, no differences between the groups were seen (Figures 2E,F). Thus, neither the release of cytotoxic granules after multiple different stimuli nor cytotoxicity was impaired in ME/CFS patients as compared to controls.”

The authors apologize for these errors and state that they do not change the scientific conclusions of the article in any way. The original article has been updated.

