# Supplementary material for: Unperturbed Cytotoxic Lymphocyte Phenotype and Function in Myalgic Encephalomyelitis/Chronic Fatigue Syndrome Patients
Source: Front Immunol. 2017 Jun 26;8:723. doi: 10.3389/fimmu.2017.00723 (PMC5483846; doi:10.3389/fimmu.2017.00723)
Supplement: Supplementary file 1 [file Table_1.PDF]

Table S1. Additional patient characteristics

			Reference population		Stockholm				Oslo						T-test for Stockholm vs Oslo patients
					Controls	Patients			Controls			Patients			
Scale	Range	Evaluated aspect	Mean	SD		Mean	SD	n	Mean	SD	n	Mean	SD	n	
Hospital anxiety and depression scale[1]	0-21	Anxiety index	3.8	3.7	-	4.2	3.8	24	4.0	2.9	24	6.7	3.6	19	0.03
	0-21	Depression index	3.1	2.8	-	6.5	3.9	24	1.0	1.5	24	4.7	3.6	19	0.14
Short form-36[2]	0-100	Physical functioning	90.9	14.5	-	44.9	22.3	24	98.7	2.2	23	37.5	24.7	18	0.32
	0-100	Role physical	84.2	30.9	-	0.2	1.0	24	97.8	7.2	23	1.4	5.9	18	0.84
	0-100	Role emotional	84.1	30.9	-	85.7	34.0	23	94.2	16.4	23	70.4	42.6	18	0.13
	0-100	Bodily Pain	76.5	25.2	-	34.0	19.9	24	85.6	17.9	23	32.8	25.0	19	0.60
	0-100	Social functioning	86.0	22.6	-	24.0	19.1	24	96.2	7.0	23	25.0	29.2	19	0.63
	0-100	Mental health	78.3	16.7	-	67.4	14.3	24	83.7	11.8	23	69.3	13.5	19	0.67
	0-100	Vitality	59.0	20.4	-	10.7	14.5	24	67.8	16.1	23	19.5	12.3	19	0.01
	0-100	Global health	80.0	21.0	-	19.1	12.1	24	88.0	11.0	23	20.9	13.8	19	0.93
						Median	Range	n	Median	Range	n	Median	Range	n	
Self reported symptoms[3]	0-4	Fatigue	-	-	-	3	1-4	22	0	0-2	16	3	2-4	18	0.50
	0-4	Concentration problems	-	-	-	2	0-4	22	0	0-2	16	4	1-4	18	0.001*
	0-4	Memory problems	-	-	-	2	0-4	22	0	0-2	17	3	1-4	18	0.01
	0-4	Fever	-	-	-	0	0-4	22	0	0-1	16	2	0-4	18	0.07
	0-4	Chills	-	-	-	2	0-4	22	0	0-3	16	3	0-4	18	0.24
	0-4	Swollen lymph nodes	-	-	-	1	0-3	22	0	0-1	16	2	0-4	17	0.07
	0-4	Joint pain	-	-	-	2	0-3	21	0.5	0-2	16	2	0-4	18	0.87
	0-4	Headache	-	-	-	2	0-4	22	1	0-3	16	2	0-4	18	0.63
	0-4	Nausea	-	-	-	2	0-4	22	0	0-2	16	1	0-4	18	0.99
	0-4	Irritable bowel	-	-	-	2	0-4	22	0	0-2	16	3	0-4	18	0.31
	0-4	Lower urinary tract symptoms	-	-	-	1	0-4	22	0	0-4	16	1.5	0-4	18	0.55

1 Calculated from Bjerkeset O. Nordahl HM, Larson S, Dahl AA, Linaker O. (2008): a 4-year follow-up study of syndromal and sub-syndromal anxiety and depression symptoms in the general population. Social Psychiatry and Psychiatric Epidemiology, 43 (3), pp 192-199.

2 Adopted from normal values for age group 30-49 years (weighted for the same female/male ratio as in this study), Jon Håvard Loge and Stein Kaasa (1998). Short Form 36 (SF-36) health survey: normative data from the general Norwegian population. Scand J Soc Med, 4, pp 250-258.

3 No refecence population data is available for the self reported symptoms

* indicates significance after Bonferroni-Holm correction for multiple comparisons
