# Supplementary material for: Unperturbed Cytotoxic Lymphocyte Phenotype and Function in Myalgic Encephalomyelitis/Chronic Fatigue Syndrome Patients
Source: Front Immunol. 2017 Jun 26;8:723. doi: 10.3389/fimmu.2017.00723 (PMC5483846; doi:10.3389/fimmu.2017.00723)
Supplement: Supplementary file 2 [file Table_2.PDF]

Table S2. Flow cytometry panels

Group	Panel name	Laser	Filter	Flourochrome	Antigen	Clone	Company	Step	Staining
Stockholm	TrueCount	B (488)	530/30	FITC	CD20	2H7	BioLegend	1	Ext
Stockholm	TrueCount	R (639)	670/30	APC	Dead cell marker	-	Invitrogen	1	Ext
Stockholm	TrueCount	R (639)	780/60	APC-Cy7	CD16	3G8	BD biosciences	1	Ext
Stockholm	TrueCount	V (405)	450/50	Pacific blue	CD57	HCD57	BioLegend	1	Ext
Stockholm	TrueCount	V (405)	570LP 585/42	Qdot565	CD8	3B5	Invitrogen	1	Ext
Stockholm	TrueCount	V (405)	710/50	Qdot705	CD4	S3.5	Invitrogen	1	Ext
Stockholm	TrueCount	YG (561)	620/14	PE-CF594	CD45	HI30	BD biosciences	1	Ext
Stockholm	TrueCount	YG (561)	710/50	PE-Cy5.5	CD3	S4.1	Invitrogen	1	Ext
Stockholm	TrueCount	YG (561)	780/60	PE-Cy7	CD56	NCAM16.2	BD biosciences	1	Ext
Stockholm	Perforin	R (639)	670/30	APC	CD8	SK1	BD Bioscience	1	Ext
Stockholm	Perforin	R (639)	730/45	AF700	CD16	3G8	BD Bioscience	1	Ext
Stockholm	Perforin	R (639)	780/60	biotin	CD57	HCD57	BioLegend	1	Ext
Stockholm	Perforin	R (639)	780/60	APC-Cy7	biotin	Streptavidin	BioLegend	2	Ext
Stockholm	Perforin	V (405)	505LP 525/50	V500	CD14	M5E2	BD Bioscience	1	Ext
Stockholm	Perforin	V (405)	505LP 525/50	V500	CD19	3G26	BD Bioscience	1	Ext
Stockholm	Perforin	V (405)	505LP 525/50	Pacific orange	Dead cell marker	3B5	Invitrogen	1	Ext
Stockholm	Perforin	V (405)	610/20	Qdot605	CD4	S3.5	Invitrogen	1	Ext
Stockholm	Perforin	V (405)	780/60	BrV785	CD3	OKT3	BioLegend	1	Ext
Stockholm	Perforin	YG (561)	586/15	PE	Perforin	dG9	BioLegend	3	Int
Stockholm	Perforin	YG (561)	780/60	PE-Cy7	CD56	NCAM16.2	BD biosciences	1	Ext
Stockholm	Granzyme A	R (639)	670/30	AF647	GrzA	CB9	BioLegend	3	Int
Stockholm	Granzyme A	R (639)	730/45	AF700	CD16	3G8	BD Bioscience	1	Ext
Stockholm	Granzyme A	R (639)	780/60	biotin	CD57	HCD57	BioLegend	1	Ext
Stockholm	Granzyme A	R (639)	780/60	APC-Cy7	biotin	Streptavidin	BioLegend	2	Ext
Stockholm	Granzyme A	V (405)	505LP 525/50	V500	CD14	M5E2	BD Bioscience	1	Ext
Stockholm	Granzyme A	V (405)	505LP 525/50	V500	CD19	3G26	BD Bioscience	1	Ext
Stockholm	Granzyme A	V (405)	505LP 525/50	Pacific orange	Dead cell marker	3B5	Invitrogen	1	Ext
Stockholm	Granzyme A	V (405)	610/20	Qdot605	CD4	S3.5	Invitrogen	1	Ext
Stockholm	Granzyme A	V (405)	710/50	Qdot705	CD8	3B5	Invitrogen	1	Ext
Stockholm	Granzyme A	V (405)	780/60	BrV785	CD3	OKT3	BioLegend	1	Ext
Stockholm	Granzyme A	YG (561)	780/60	PE-Cy7	CD56	NCAM16.2	BD biosciences	1	Ext
Stockholm	Granzyme A isotype	R (639)	670/30	AF647	Isotype	MOPC-21	BioLegend	3	Int
Stockholm	Granzyme A isotype	R (639)	730/45	AF700	CD16	3G8	BD Bioscience	1	Ext
Stockholm	Granzyme A isotype	R (639)	780/60	biotin	CD57	HCD57	BioLegend	1	Ext
Stockholm	Granzyme A isotype	R (639)	780/60	APC-Cy7	biotin	Streptavidin	BioLegend	2	Ext
Stockholm	Granzyme A isotype	V (405)	450/50	Pacific blue	Isotype	MOPC-21	BioLegend	3	Int
Stockholm	Granzyme A isotype	V (405)	505LP 525/50	V500	CD14	M5E2	BD Bioscience	1	Ext
Stockholm	Granzyme A isotype	V (405)	505LP 525/50	V500	CD19	3G26	BD Bioscience	1	Ext
Stockholm	Granzyme A isotype	V (405)	505LP 525/50	Pacific orange	Dead cell marker	3B5	Invitrogen	1	Ext
Stockholm	Granzyme A isotype	V (405)	610/20	Qdot605	CD4	S3.5	Invitrogen	1	Ext
Stockholm	Granzyme A isotype	V (405)	670/30	Qdot655	CD45RA	MEM-56	Invitrogen	1	Ext
Stockholm	Granzyme A isotype	V (405)	710/50	Qdot705	CD8	3B5	Invitrogen	1	Ext
Stockholm	Granzyme A isotype	V (405)	780/60	BrV785	CD3	OKT3	BioLegend	1	Ext
Stockholm	Granzyme A isotype	YG (561)	780/60	PE-Cy7	CD56	NCAM16.2	BD biosciences	1	Ext
Stockholm	Granzyme B	R (639)	780/60	APC-Cy7	CD16	3G8	BD biosciences	1	Ext
Stockholm	Granzyme B	V (405)	450/50	Pacific blue	CD57	HCD57	BioLegend	1	Ext
Stockholm	Granzyme B	V (405)	505LP 525/50	V500	CD14	M5E2	BD Bioscience	1	Ext
Stockholm	Granzyme B	V (405)	505LP 525/50	V500	CD19	3G26	BD Bioscience	1	Ext
Stockholm	Granzyme B	V (405)	505LP 525/50	Pacific orange	Dead cell marker	3B5	Invitrogen	1	Ext
Stockholm	Granzyme B	V (405)	670/30	Qdot655	CD4	S3.5	Invitrogen	1	Ext
Stockholm	Granzyme B	V (405)	710/50	Qdot705	CD8	3B5	Invitrogen	1	Ext
Stockholm	Granzyme B	YG (561)	620/14	PE-CF594	Granzyme B	GB11	BD horizon	2	Int
Stockholm	Granzyme B	YG (561)	710/50	PE-Cy5.5	CD3	S4.1	Invitrogen	1	Ext
Stockholm	Granzyme B	YG (561)	780/60	PE-Cy7	CD56	NCAM16.2	BD biosciences	1	Ext
Stockholm	Granzyme B isotype	R (639)	780/60	APC-Cy7	CD16	3G8	BD biosciences	1	Ext
Stockholm	Granzyme B isotype	V (405)	450/50	Pacific blue	CD57	HCD57	BioLegend	1	Ext
Stockholm	Granzyme B isotype	V (405)	505LP 525/50	V500	CD14	M5E2	BD Bioscience	1	Ext
Stockholm	Granzyme B isotype	V (405)	505LP 525/50	V500	CD19	3G26	BD Bioscience	1	Ext
Stockholm	Granzyme B isotype	V (405)	505LP 525/50	Pacific orange	Dead cell marker	3B5	Invitrogen	1	Ext
Stockholm	Granzyme B isotype	V (405)	670/30	Qdot655	CD4	S3.5	Invitrogen	1	Ext
Stockholm	Granzyme B isotype	V (405)	710/50	Qdot705	CD8	3B5	Invitrogen	1	Ext
Stockholm	Granzyme B isotype	YG (561)	620/14	PE-CF594	Isotype	Not specified	BD horizon	2	Int
Stockholm	Granzyme B isotype	YG (561)	710/50	PE-Cy5.5	CD3	S4.1	Invitrogen	1	Ext
Stockholm	Granzyme B isotype	YG (561)	780/60	PE-Cy7	CD56	NCAM16.2	BD biosciences	1	Ext

Group	Panel name	Laser	Filter	Flourochrome	Antigen	Clone	Company	Step	Staining
Oslo	Cytotoxic proteins 1	B (488)	530/30	FITC	GrzB	GB11	BioLegend	3	Int
Oslo	Cytotoxic proteins 1	B (488)	670/30	PerCP	CD3	UCHT1	BioLegend	1	Ext
Oslo	Cytotoxic proteins 1	R (639)	670/30	AF647	FcER1g	Polyclonal rabbit	Merck Millipore	3	Int
Oslo	Cytotoxic proteins 1	R (639)	730/45	AF700	GrzA	CB9	BioLegend	3	Int
Oslo	Cytotoxic proteins 1	R (639)	780/60	mouse-IgM	CD57	HCD57	BioLegend	1	Ext
Oslo	Cytotoxic proteins 1	R (639)	780/60	APC-Cy7	mouse-IgM	RMM-1	BioLegend	2	Ext
Oslo	Cytotoxic proteins 1	V (405)	505LP 525/50	V500	CD14	M5E2	BD Bioscience	1	Ext
Oslo	Cytotoxic proteins 1	V (405)	505LP 525/50	V500	CD19	3G26	BD Bioscience	1	Ext
Oslo	Cytotoxic proteins 1	V (405)	505LP 525/50	Pacific orange	Dead cell marker	3B5	Invitrogen	1	Ext
Oslo	Cytotoxic proteins 1	V (405)	610/20	biotin	CD16	3G8	BD Bioscience	1	Ext
Oslo	Cytotoxic proteins 1	V (405)	610/20	Qdot605	biotin	Streptavidin	Invitrogen	2	Ext
Oslo	Cytotoxic proteins 1	V (405)	670/30	Qdot655	CD4	S3.5	Invitrogen	1	Ext
Oslo	Cytotoxic proteins 1	V (405)	710/50	Qdot705	CD8	3B5	Invitrogen	1	Ext
Oslo	Cytotoxic proteins 1	V (405)	780/60	BrV785	CD45RA	HI100	BioLegend	1	Ext
Oslo	Cytotoxic proteins 1	YG (561)	586/15	PE	Perforin	dG9	BioLegend	3	Int
Oslo	Cytotoxic proteins 1	YG (561)	620/14	ECD	CD69	TP1.55.3	Beckman Coulter	1	Ext
Oslo	Cytotoxic proteins 1	YG (561)	710/50	PE-Cy5.5	TCR-gamma/delta	IMMU510	Beckman Coulter	1	Ext
Oslo	Cytotoxic proteins 1	YG (561)	780/60	PE-Cy7	CD56	NCAM16.2	BD biosciences	1	Ext
Oslo	Cytotoxic proteins 1 isotype	B (488)	530/30	FITC	Isotype	MOPC-21	BD Bioscience	3	Int
Oslo	Cytotoxic proteins 1 sotype	B (488)	670/30	PerCP	CD3	UCHT1	BioLegend	1	Ext
Oslo	Cytotoxic proteins 1 sotype	R (639)	670/30	AF647	FcER1g	Polyclonal rabbit	Merck Millipore	3	Int
Oslo	Cytotoxic proteins 1 sotype	R (639)	730/45	AF700	Isotype	MOPC-21	BioLegend	3	Int
Oslo	Cytotoxic proteins 1 sotype	R (639)	780/60	mouse-IgM	CD57	HCD57	BioLegend	1	Ext
Oslo	Cytotoxic proteins 1 sotype	R (639)	780/60	APC-Cy7	mouse-IgM	RMM-1	BioLegend	2	Ext
Oslo	Cytotoxic proteins 1 sotype	V (405)	505LP 525/50	V500	CD14	M5E2	BD Bioscience	1	Ext
Oslo	Cytotoxic proteins 1 sotype	V (405)	505LP 525/50	V500	CD19	3G26	BD Bioscience	1	Ext
Oslo	Cytotoxic proteins 1 sotype	V (405)	505LP 525/50	Pacific orange	Dead cell marker	3B5	Invitrogen	1	Ext
Oslo	Cytotoxic proteins 1 sotype	V (405)	610/20	biotin	CD16	3G8	BD Bioscience	1	Ext
Oslo	Cytotoxic proteins 1 sotype	V (405)	610/20	Qdot605	biotin	Streptavidin	Invitrogen	2	Ext
Oslo	Cytotoxic proteins 1 sotype	V (405)	670/30	Qdot655	CD4	S3.5	Invitrogen	1	Ext
Oslo	Cytotoxic proteins 1 sotype	V (405)	710/50	Qdot705	CD8	3B5	Invitrogen	1	Ext
Oslo	Cytotoxic proteins 1 sotype	V (405)	780/60	BrV785	CD45RA	HI100	BioLegend	1	Ext
Oslo	Cytotoxic proteins 1 sotype	YG (561)	586/15	PE	Isotype	27-35	BD Bioscience	3	Int
Oslo	Cytotoxic proteins 1 sotype	YG (561)	620/14	ECD	CD69	TP1.55.3	Beckman Coulter	1	Ext
Oslo	Cytotoxic proteins 1 sotype	YG (561)	710/50	PE-Cy5.5	TCR-gamma/delta	IMMU510	Beckman Coulter	1	Ext
Oslo	Cytotoxic proteins 1 sotype	YG (561)	780/60	PE-Cy7	CD56	NCAM16.2	BD biosciences	1	Ext
Oslo	Cytotoxic proteins 2: CD57	R (639)	670/30	AF647	Granzyme A	CB9	BioLegend	3	Int
Oslo	Cytotoxic proteins 2: CD57	R (639)	780/60	mouse-IgM	CD57	HCD57	BioLegend	1	Ext
Oslo	Cytotoxic proteins 2: CD57	R (639)	780/60	APC-Cy7	mouse-IgM	RMM-1	BioLegend	2	Ext
Oslo	Cytotoxic proteins 2: CD57	V (405)	505LP 525/50	V500	CD14	M5E2	BD Bioscience	1	Ext
Oslo	Cytotoxic proteins 2: CD57	V (405)	505LP 525/50	V500	CD19	3G26	BD Bioscience	1	Ext
Oslo	Cytotoxic proteins 2: CD57	V (405)	505LP 525/50	Pacific orange	Dead cell marker	3B5	Invitrogen	1	Ext
Oslo	Cytotoxic proteins 2: CD57	V (405)	670/30	biotin	TCR-gd	B1	BioLegend	1	Ext
Oslo	Cytotoxic proteins 2: CD57	V (405)	670/30	Qdot655	biotin	Streptavidin	Invitrogen	2	Ext
Oslo	Cytotoxic proteins 2: CD57	V (405)	710/50	Qdot705	CD8	3B5	Invitrogen	1	Ext
Oslo	Cytotoxic proteins 2: CD57	V (405)	780/60	BrV785	CD3	OKT3	BioLegend	1	Ext
Oslo	Cytotoxic proteins 2: CD57	YG (561)	586/15	PE	Perforin	dG9	BioLegend	3	Int
Oslo	Cytotoxic proteins 2: CD57	UV (305)	740/35	BUV737	CD4	SK3	BD biosciences	1	Ext
Stockholm	CD69	R (639)	730/45	AF700	CD16	3G8	BD Bioscience	1	Ext
Stockholm	CD69	R (639)	780/60	biotin	CD57	HCD57	BioLegend	1	Ext
Stockholm	CD69	R (639)	780/60	APC-Cy7	biotin	Streptavidin	BioLegend	2	Ext
Stockholm	CD69	V (405)	505LP 525/50	V500	CD14	M5E2	BD Bioscience	1	Ext
Stockholm	CD69	V (405)	505LP 525/50	V500	CD19	3G26	BD Bioscience	1	Ext
Stockholm	CD69	V (405)	505LP 525/50	Pacific orange	Dead cell marker	3B5	Invitrogen	1	Ext
Stockholm	CD69	V (405)	610/20	Qdot605	CD4	S3.5	Invitrogen	1	Ext
Stockholm	CD69	V (405)	710/50	Qdot705	CD8	3B5	Invitrogen	1	Ext
Stockholm	CD69	V (405)	780/60	BrV785	CD3	OKT3	BioLegend	1	Ext
Stockholm	CD69	YG (561)	620/14	ECD	CD69	TP1.55.3	Beckman Coulter	1	Ext
Stockholm	CD69	YG (561)	780/60	PE-Cy7	CD56	NCAM16.2	BD biosciences	1	Ext
Oslo	CD69	B (488)	670/30	PerCP	CD3	UCHT1	BioLegend	1	Ext
Oslo	CD69	R (639)	730/45	AF700	GrzA	CB9	BioLegend	3	Int
Oslo	CD69	R (639)	780/60	mouse-IgM	CD57	HCD57	BioLegend	1	Ext
Oslo	CD69	R (639)	780/60	APC-Cy7	mouse-IgM	RMM-1	BioLegend	2	Ext
Oslo	CD69	V (405)	505LP 525/50	V500	CD14	M5E2	BD Bioscience	1	Ext
Oslo	CD69	V (405)	505LP 525/50	V500	CD19	3G26	BD Bioscience	1	Ext
Oslo	CD69	V (405)	505LP 525/50	Pacific orange	Dead cell marker	3B5	Invitrogen	1	Ext
Oslo	CD69	V (405)	610/20	biotin	CD16	3G8	BD Bioscience	1	Ext
Oslo	CD69	V (405)	610/20	Qdot605	biotin	Streptavidin	Invitrogen	2	Ext
Oslo	CD69	V (405)	670/30	Qdot655	CD4	S3.5	Invitrogen	1	Ext
Oslo	CD69	V (405)	710/50	Qdot705	CD8	3B5	Invitrogen	1	Ext
Oslo	CD69	YG (561)	620/14	ECD	CD69	TP1.55.3	Beckman Coulter	1	Ext
Oslo	CD69	YG (561)	780/60	PE-Cy7	CD56	NCAM16.2	BD biosciences	1	Ext

Group	Panel name	Laser	Filter	Flourochrome	Antigen	Clone	Company	Step	Staining
Stockholm	Activation/Ki67	B (488)	530/30	AF488	CD3	SK7	BD Bioscience	1	Ext
Stockholm	Activation/Ki67	R (639)	670/30	APC	PD-1	EH12.2H7	BioLegend	1	Ext
Stockholm	Activation/Ki67	R (639)	730/45	AF700	CD16	3G8	BD Bioscience	1	Ext
Stockholm	Activation/Ki67	R (639)	780/60	biotin	CD57	HCD57	BioLegend	1	Ext
Stockholm	Activation/Ki67	R (639)	780/60	APC-Cy7	biotin	Streptavidin	BioLegend	2	Ext
Stockholm	Activation/Ki67	V (405)	505LP 525/50	V500	CD14	M5E2	BD Bioscience	1	Ext
Stockholm	Activation/Ki67	V (405)	505LP 525/50	V500	CD19	3G26	BD Bioscience	1	Ext
Stockholm	Activation/Ki67	V (405)	505LP 525/50	Pacific orange	Dead cell marker	3B5	Invitrogen	1	Ext
Stockholm	Activation/Ki67	V (405)	610/20	Qdot605	CD4	S3.5	Invitrogen	1	Ext
Stockholm	Activation/Ki67	V (405)	670/30	Qdot655	CD45RA	MEM-56	Invitrogen	1	Ext
Stockholm	Activation/Ki67	V (405)	710/50	Qdot705	CD8	3B5	Invitrogen	1	Ext
Stockholm	Activation/Ki67	V (405)	780/60	BrV785	HLA-DR	L243	BioLegend	1	Ext
Stockholm	Activation/Ki67	YG (561)	586/15	PE	Ki67	Ki-67	BioLegend	3	Int
Stockholm	Activation/Ki67	YG (561)	620/14	ECD	CD38	LS198.4.3	Beckman Coulter	1	Ext
Stockholm	Activation/Ki67	YG (561)	710/50	PE-Cy5.5	TCR-gamma/delta	IMMU510	Beckman Coulter	1	Ext
Stockholm	Activation/Ki67	YG (561)	780/60	PE-Cy7	CD56	NCAM16.2	BD Bioscience	1	Ext
Oslo	Activation	R (639)	730/45	AF700	CD16	3G8	BD Bioscience	1	Ext
Oslo	Activation	R (639)	780/60	mouse-IgM	CD57	HCD57	BioLegend	1	Ext
Oslo	Activation	R (639)	780/60	APC-Cy7	CD3	HIT3a	BioLegend	2	Ext
Oslo	Activation	V (405)	450/50	Pacific blue	CD57	HCD57	BioLegend	1	Ext
Oslo	Activation	V (405)	505LP 525/50	V500	CD4	RPA-T4	BD Bioscience	1	Ext
Oslo	Activation	V (405)	505LP 525/50	V500	CD14	M5E2	BD Bioscience	1	Ext
Oslo	Activation	V (405)	505LP 525/50	V500	CD19	3G26	BD Bioscience	1	Ext
Oslo	Activation	V (405)	505LP 525/50	Pacific orange	Dead cell marker	3B5	Invitrogen	1	Ext
Oslo	Activation	V (405)	670/30	biotin	TCR-gd	B1	BioLegend	2	Int
Oslo	Activation	V (405)	670/30	Qdot655	Streptavidin	-	Invitrogen	3	Int
Oslo	Activation	V (405)	710/50	Qdot705	CD8	3B5	Invitrogen	1	Ext
Oslo	Activation	V (405)	780/60	BV786	CD38	HIT2	BD Bioscience	1	Ext
Oslo	Activation	YG (561)	586/15	PE	HLA-DR	Tu36	BioLegend	1	Ext
Oslo	Activation	UV (305)	379/28	BUV395	CD56	NCAM16.2	BD Bioscience	1	Ext
Oslo	Activation	UV (305)	740/35	BUV737	CD279	EH12.1	BD Bioscience	1	Ext
Stockholm	Adaptive expansions	B (488)	530/30	AF488	FcER1g	Polyclonal	Millipore	3	Int
Stockholm	Adaptive expansions	R (639)	670/30	APC	SYK	4D10	BD Bioscience	3	Int
Stockholm	Adaptive expansions	R (639)	730/45	AF700	CD16	3G8	BD Bioscience	1	Ext
Stockholm	Adaptive expansions	R (639)	780/60	biotin	CD57	HCD57	BioLegend	1	Ext
Stockholm	Adaptive expansions	R (639)	780/60	APC-Cy7	biotin	Streptavidin	BioLegend	2	Ext
Stockholm	Adaptive expansions	V (405)	450/50	Rabbit	EAT-2	polyclonal rabbit	Proteintech Group	3	Int
Stockholm	Adaptive expansions	V (405)	450/50	Pacific blue	anti-Rabbit	polyclonal goat	Invitrogen	4	Int
Stockholm	Adaptive expansions	V (405)	505LP 525/50	V500	CD14	M5E2	BD Bioscience	1	Ext
Stockholm	Adaptive expansions	V (405)	505LP 525/50	V500	CD19	3G26	BD Bioscience	1	Ext
Stockholm	Adaptive expansions	V (405)	505LP 525/50	Pacific orange	Dead cell marker	3B5	Invitrogen	1	Ext
Stockholm	Adaptive expansions	V (405)	610/20	Qdot605	CD4	S3.5	Invitrogen	1	Ext
Stockholm	Adaptive expansions	V (405)	710/50	Qdot705	CD8	3B5	Invitrogen	1	Ext
Stockholm	Adaptive expansions	V (405)	780/60	BrV785	CD3	OKT3	BioLegend	1	Ext
Stockholm	Adaptive expansions	YG (561)	586/15	PE	PLZF	Mags.21F7	eBioscience	3	Int
Stockholm	Adaptive expansions	YG (561)	780/60	PE-Cy7	CD56	NCAM16.2	BD Bioscience	1	Ext
Stockholm	NKG2C	R (639)	730/45	AF700	CD16	3G8	BD Bioscience	1	Ext
Stockholm	NKG2C	R (639)	780/60	mouse-IgM	CD57	HCD57	BioLegend	1	Ext
Stockholm	NKG2C	R (639)	780/60	APC-Cy7	mouse-IgM	RMM-1	BioLegend	2	Ext
Stockholm	NKG2C	V (405)	505LP 525/50	V500	CD14	M5E2	BD Bioscience	1	Ext
Stockholm	NKG2C	V (405)	505LP 525/50	V500	CD19	3G26	BD Bioscience	1	Ext
Stockholm	NKG2C	V (405)	505LP 525/50	Pacific orange	Dead cell marker	3B5	Invitrogen	1	Ext
Stockholm	NKG2C	V (405)	610/20	Qdot605	CD4	S3.5	Invitrogen	1	Ext
Stockholm	NKG2C	V (405)	670/30	Qdot655	biotin	Streptavidin	Invitrogen	2	Ext
Stockholm	NKG2C	V (405)	710/50	Qdot705	CD8	3B5	Invitrogen	1	Ext
Stockholm	NKG2C	YG (561)	586/15	PE	NKG2C	134591	R&D Systems	1	Ext
Stockholm	NKG2C	YG (561)	710/50	PE-Cy5.5	CD3	S4.1	Invitrogen	1	Ext
Stockholm	NKG2C	YG (561)	780/60	PE-Cy7	CD56	NCAM16.2	BD Bioscience	1	Ext
Oslo	Adaptive expansions	B (488)	530/30	AF488	FcER1g	Polyclonal	Millipore	5	Int
Oslo	Adaptive expansions	B (488)	670/30	PerCP	CD3	UCHT1	BioLegend	1	Ext
Oslo	Adaptive expansions	R (639)	670/30	APC	SYK	4D10	BD Bioscience	5	Int
Oslo	Adaptive expansions	R (639)	730/45	AF700	CD16	3G8	BD Bioscience	1	Ext
Oslo	Adaptive expansions	R (639)	780/60	mouse-IgM	CD57	HCD57	BioLegend	1	Ext
Oslo	Adaptive expansions	R (639)	780/60	APC-Cy7	mouse-IgM	RMM-1	BioLegend	2	Ext
Oslo	Adaptive expansions	V (405)	450/50	Rabbit	EAT-2	polyclonal rabbit	Proteintech Group	3	Int
Oslo	Adaptive expansions	V (405)	450/50	Pacific blue	anti-Rabbit	polyclonal goat	Invitrogen	4	Int
Oslo	Adaptive expansions	V (405)	505LP 525/50	V500	CD14	M5E2	BD Bioscience	1	Ext
Oslo	Adaptive expansions	V (405)	505LP 525/50	V500	CD19	3G26	BD Bioscience	1	Ext
Oslo	Adaptive expansions	V (405)	505LP 525/50	Pacific orange	Dead cell marker	3B5	Invitrogen	1	Ext
Oslo	Adaptive expansions	V (405)	610/20	biotin	NKG2C	REA205	Miltenyi	1	Ext
Oslo	Adaptive expansions	V (405)	610/20	Qdot605	biotin	Streptavidin	Invitrogen	2	Ext
Oslo	Adaptive expansions	V (405)	670/30	Qdot655	CD4	S3.5	Invitrogen	1	Ext
Oslo	Adaptive expansions	V (405)	710/50	Qdot705	CD8	3B5	Invitrogen	1	Ext
Oslo	Adaptive expansions	V (405)	780/60	BrV785	CD45RA	HI100	BioLegend	1	Ext
Oslo	Adaptive expansions	YG (561)	586/15	PE	PLZF	Mags.21F7	eBioscience	5	Int
Oslo	Adaptive expansions	YG (561)	620/14	PE-TxR	CD2	S5.5	Invitrogen	1	Ext
Oslo	Adaptive expansions	YG (561)	710/50	PE-Cy5.5	TCR-gamma/delta	IMMU510	Beckman Coulter	1	Ext
Oslo	Adaptive expansions	YG (561)	780/60	PE-Cy7	CD56	NCAM16.2	BD Bioscience	1	Ext

Group	Panel name	Laser	Filter	Flourochrome	Antigen	Clone	Company	Step	Staining
Stockholm	Functional	B (488)	530/30	Cell Tracker Green	Bar code	-	Invitrogen	1	Before stim
Stockholm	Functional	R (639)	670/30	APC	CD4	SK3	BD Bioscience	2	Ext
Stockholm	Functional	R (639)	780/60	AF700	TNF	MAb11	BD Bioscience	4	Int
Stockholm	Functional	R (639)	780/60	APC-AF750	CD3	S4.1	Invitrogen	2	Ext
Stockholm	Functional	V (405)	450/50	Pacific blue	CD107a	H4A3	BioLegend	2	Ext
Stockholm	Functional	V (405)	505LP 525/50	V500	CD14	M5E2	BD Bioscience	2	Ext
Stockholm	Functional	V (405)	505LP 525/50	V500	CD19	3G26	BD Bioscience	2	Ext
Stockholm	Functional	V (405)	505LP 525/50	Pacific orange	Dead cell marker	3B5	Invitrogen	2	Ext
Stockholm	Functional	V (405)	610/20	BV605	IFN-g	B27	BD Bioscience	4	Int
Stockholm	Functional	V (405)	670/30	Qdot655	CD57	HCD57	BioLegend	2	Ext
Stockholm	Functional	V (405)	670/30	eFluor650NC	mouse-IgM	II/41	eBioscience	3	Ext
Stockholm	Functional	V (405)	710/50	Qdot705	CD8	3B5	Invitrogen	2	Ext
Stockholm	Functional	YG (561)	710/50	PE-Cy5.5	TCR-gamma/delta	IMMU510	Beckman Coulter	2	Ext
Stockholm	Functional	YG (561)	780/60	PE-Cy7	CD56	NCAM16.2	BD Bioscience	2	Ext
Oslo	Functional 1: T-cells	B (488)	530/30	Cell Tracker Green	Bar code	-	Invitrogen	1	Before stim
Oslo	Functional 1: T-cells	B (488)	670/30	PerCP	CD3	UCHT1	BioLegend	2	Ext
Oslo	Functional 1: T-cells	R (639)	730/45	AF700	IFN-g	B27	BD Bioscience	2	Int
Oslo	Functional 1: T-cells	R (639)	780/60	mouse-IgM	CD57	HCD57	BioLegend	2	Ext
Oslo	Functional 1: T-cells	R (639)	780/60	APC-Cy7	mouse-IgM	RMM-1	BioLegend	3	Ext
Oslo	Functional 1: T-cells	V (405)	450/50	Pacific blue	CD107a	H4A3	BioLegend	2	Ext
Oslo	Functional 1: T-cells	V (405)	505LP 525/50	V500	CD14	M5E2	BD Bioscience	2	Ext
Oslo	Functional 1: T-cells	V (405)	505LP 525/50	V500	CD19	3G26	BD Bioscience	2	Ext
Oslo	Functional 1: T-cells	V (405)	505LP 525/50	Pacific orange	Dead cell marker	3B5	Invitrogen	2	Ext
Oslo	Functional 1: T-cells	V (405)	610/20	biotin	TNF	Mab11	BD Bioscience	2	Ext
Oslo	Functional 1: T-cells	V (405)	610/20	Qdot605	biotin	Streptavidin	Invitrogen	5	Int
Oslo	Functional 1: T-cells	V (405)	670/30	Qdot655	CD4	MEM-56	Invitrogen	2	Ext
Oslo	Functional 1: T-cells	V (405)	710/50	Qdot705	CD8	3B5	Invitrogen	2	Ext
Oslo	Functional 1: T-cells	V (405)	780/60	BrV785	CD3	OKT3	BioLegend	2	Ext
Oslo	Functional 1: T-cells	YG (561)	710/50	PE-Cy5.5	TCR-gamma/delta	IMMU510	Beckman Coulter	2	Ext
Oslo	Functional 1: T-cells	YG (561)	780/60	PE-Cy7	CD56	NCAM16.2	BD Bioscience	2	Ext
Stockholm	Adrenaline inhibition	B (488)	530/30	Cell Tracker Green	Bar code	-	Invitrogen	1	Before stim
Stockholm	Adrenaline inhibition	R (639)	670/30	APC	IFN-g	B27	BD Bioscience	2	Ext
Stockholm	Adrenaline inhibition	R (639)	730/45	AF700	TNF	MAb11	BD Bioscience	4	Int
Stockholm	Adrenaline inhibition	R (639)	780/60	biotin	CD57	HCD57	BioLegend	2	Ext
Stockholm	Adrenaline inhibition	R (639)	780/60	APC-Cy7	biotin	Streptavidin	BioLegend	3	Ext
Stockholm	Adrenaline inhibition	V (405)	450/50	Pacific blue	CD107a	H4A3	BioLegend	2	Ext
Stockholm	Adrenaline inhibition	V (405)	505LP 525/50	V500	CD14	M5E2	BD Bioscience	2	Ext
Stockholm	Adrenaline inhibition	V (405)	505LP 525/50	V500	CD19	3G26	BD Bioscience	2	Ext
Stockholm	Adrenaline inhibition	V (405)	505LP 525/50	Pacific orange	Dead cell marker	3B5	Invitrogen	2	Ext
Stockholm	Adrenaline inhibition	V (405)	610/20	Qdot605	CD4	S3.5	Invitrogen	2	Ext
Stockholm	Adrenaline inhibition	V (405)	710/50	Qdot705	CD8	3B5	Invitrogen	2	Ext
Stockholm	Adrenaline inhibition	V (405)	780/60	BrV785	CD3	OKT3	BioLegend	2	Ext
Stockholm	Adrenaline inhibition	YG (561)	710/50	PE-Cy5.5	TCR-gamma/delta	IMMU510	Beckman Coulter	2	Ext
Stockholm	Adrenaline inhibition	YG (561)	780/60	PE-Cy7	CD56	NCAM16.2	BD Bioscience	2	Ext
Oslo	Functional 2: NK-cells	B (488)	530/30	FITC	CD8	SK1	BD Bioscience	1	Ext
Oslo	Functional 2: NK-cells	R (639)	670/30	APC	IFN-g	B27	BD Bioscience	2	Int
Oslo	Functional 2: NK-cells	V (405)	450/50	Pacific blue	CD57	HCD57	BioLegend	1	Ext
Oslo	Functional 2: NK-cells	V (405)	505LP 525/50	V500	CD14	M5E2	BD Bioscience	1	Ext
Oslo	Functional 2: NK-cells	V (405)	505LP 525/50	V500	CD19	3G26	BD Bioscience	1	Ext
Oslo	Functional 2: NK-cells	V (405)	505LP 525/50	Pacific orange	Dead cell marker	3B5	Invitrogen	1	Ext
Oslo	Functional 2: NK-cells	V (405)	610/20	Qdot605	CD4	MEM-56	Invitrogen	1	Ext
Oslo	Functional 2: NK-cells	V (405)	780/60	BrV785	CD3	OKT3	BioLegend	1	Ext
Oslo	Functional 2: NK-cells	YG (561)	586/15	PE	TNF	Mab11	BioLegend	2	Int
Oslo	Functional 2: NK-cells	YG (561)	780/60	PE-Cy7	CD56	NCAM16.2	BD Bioscience	1	Ext
Oslo	Functional 2: NK-cells	UV (305)	379/28	BUV395	CD107a	H4A3	BD Bioscience	1	Ext
