# Supplementary material for: Unperturbed Cytotoxic Lymphocyte Phenotype and Function in Myalgic Encephalomyelitis/Chronic Fatigue Syndrome Patients
Source: Front Immunol. 2017 Jun 26;8:723. doi: 10.3389/fimmu.2017.00723 (PMC5483846; doi:10.3389/fimmu.2017.00723)
Supplement: Supplementary file 3 [file Table_3.PDF]

Table S3. Variance explained by individual principal components

Principal component	Stockholm variance		Oslo variance	
	Explained	Cumulative	Explained	Cumulative
1	0.15	0.15	0.20	0.20
2	0.10	0.24	0.17	0.37
3	0.09	0.33	0.09	0.46
4	0.08	0.41	0.07	0.53
5	0.07	0.48	0.05	0.58
6	0.06	0.54	0.05	0.63
7	0.05	0.59	0.04	0.68
8	0.04	0.64	0.04	0.71
9	0.04	0.67	0.04	0.75
10	0.03	0.71	0.03	0.78
11	0.03	0.74	0.02	0.80
12	0.03	0.76	0.02	0.83
13	0.02	0.79	0.02	0.85
14	0.02	0.81	0.02	0.86
