# Supplementary material for: Unperturbed Cytotoxic Lymphocyte Phenotype and Function in Myalgic Encephalomyelitis/Chronic Fatigue Syndrome Patients
Source: Front Immunol. 2017 Jun 26;8:723. doi: 10.3389/fimmu.2017.00723 (PMC5483846; doi:10.3389/fimmu.2017.00723)
Supplement: Supplementary file 4 [file Table_4.PDF]

Table S4. Relevant hits from Pubmed search using: ("Fatigue Syndrome, Chronic"[Mesh]) AND "Killer Cells, Natural"[Mesh]

Studies including baseline killing assays	First author	Last author	Year	Criteria	n patients (% females)	n controls (% females)	Age patients	Age controls	Age statistic	Effectors	Target cell	Perforin	Impaired cytotoxicity
Immunologic abnormalities in chronic fatigue syndrome.	Klimas NG	Fletcher MA	1990	Holmes	30 (60)	86 (47)	26-70	18-65	Range	Whole blood	K562	No	Yes
Psychobehavioral and immunological characteristics of adult people with chronic fatigue and patients with chronic fatigue	Masuda A	Tanaka H.	1994	Holmes	10(60)	21(19)	34	46	Mean	Not clear	K562	No	Yes
α-Interferon treatment of patients with chronic fatigue syndrome.	See DM	Tilles JG	1994	Holmes	30(80)	20(55)	37	23-34	Mean, range	Fresh PBMC*	K562	No	Yes
Immune responses associated with chronic fatigue syndrome: a case-control study.	Mawle AC	Reeves WC	1997	Holmes	26 (88)	50 (Matched)	Matched	Matched	-	Fresh PBMC*	K562	No	No
In vitro effects of echinacea and ginseng on natural killer and antibody- dependent cell cytotoxicity in healthy subjects and chronic	See DM	Tilles JG	1997	Holmes	20(75)	20 (55)	36	35	Mean	Fresh PBMC*	K562	No	Yes
Decreased nitric oxide-mediated natural killer cell activation in chronic fatigue syndrome.	Ogawa M	Matsuzawa Y	1998	Holmes	20(40)	21(33)	19-34	22-39	Range	Fresh/24h incubated PBMC	K562	No	No
The in vitro immunomodulatory effects of glyconutrients on peripheral blood mononuclear cells of patients with chronic fatigue syndrome.	See DM	Tilles J	1998	Holmes+ lab criteria	212 (Matched)	30 (Matched)	Matched	Matched	-	Fresh PBMC*	Herpes infected cell line	No	Yes
Psychosocial characteristics and immunological functions in patients with postinfectious chronic fatigue syndrome and noninfectious chronic fatigue syndrome.	Masuda A	Tei C	2002	Schluenderberg +infection	16 (62)	20 (70)	26	31	Mean	Not clear	K562	No	Yes
Chronic fatigue syndrome is associated with diminished intracellular perforin.	Maher KJ	Fletcher MA	2005	Fukuda	30 (83)	19 (84)	46	43	Mean	Whole blood	K562	Yes	Yes
Biomarkers in chronic fatigue syndrome: evaluation of natural killer cell function and dipeptidyl peptidase IV/CD26.	Fletcher MA	Klimas NG	2010	Fukuda	176(83)	230(86)	44	41	Mean	Whole blood	K562	No	Yes
Immune and hemorheological changes in chronic fatigue syndrome.	Brenu EW	Marshall-Gradisnik SM	2010	Fukuda	10(?)	10(?)	?	?	-	Fresh PBMC*	K562	No	Yes
Immunological abnormalities as potential biomarkers in Chronic Fatigue Syndrome/Myalgic Encephalomyelitis.	Brenu EW	Marshall-Gradisnik SM	2011	Fukuda	95 (71)	50 (58)	46	42	Mean	Fresh PBMC*	K562	Not protein	Yes
Longitudinal investigation of natural killer cells and cytokines in chronic fatigue syndrome/myalgic encephalomyelitis.	Brenu EW	Marshall-Gradisnik SM	2012	Fukuda	65 (75)	21 (66)	47	45	Mean	Fresh PBMC*	K562	No	Yes
Role of adaptive and innate immune cells in chronic fatigue syndrome/myalgic encephalomyelitis.	Brenu EW	Marshall-Gradisnik SM	2014	Fukuda	30(?)	25 (?)	51	50	Mean	Fresh PBMC*	K562	Yes	Yes

Phenotypic studies	First author	Last author	Year	Criteria	n patients (% females)	n controls (% females)	Age patients	Age controls	Age statistic	Perforin	Aberrant phenotype
Changes in immune parameters seen in Gulf War veterans but not in civilians with chronic fatigue syndrome.	Zhang Q	Natelson BH	1999	Fukuda	68(79)	53(87)	Matched	Matched	-	No	No
Predictive immunophenotypes: disease-related profile in chronic fatigue syndrome.	Stewart CC	Warner CL	2003	Holmes	90 (69)	50 (76)	37	36	Mean	No	Mixed
Lymphocyte subset differences in patients with chronic fatigue syndrome, multiple sclerosis and major depression.	Robertson MJ	Komaroff AL	2005	Holmes	23(74)	25(76)	39	38	Mean	No	Yes
Screening NK-, B- and T-cell phenotype and function in patients suffering from Chronic Fatigue Syndrome.	Curriu M	Blanco J	2013	Fukuda	22(73)	30(55)	44	38	Mean	No	Yes
Characterisation of cell functions and receptors in Chronic Fatigue Syndrome/Myalgic Encephalomyelitis (CFS/ME).	Hardcastle SL	Marshall-Gradisnik SM	2015	Fukuda	27 (78)	18 (67)	44	40	Mean	Yes (only data for non-NK-cells shown)	Yes
Pilot Study of Natural Killer Cells in Chronic Fatigue Syndrome/Myalgic Encephalomyelitis and Multiple Sclerosis.	Huth TK	Marshall-Gradisnik SM	2016	ICC 2011	14(100)	19(100)	49	48	Mean	Yes	Yes

*"Fresh PBMC" indicates that the cells have been used immediately after isolation

[illegible] abstract
