# Supplementary material for: Unperturbed Cytotoxic Lymphocyte Phenotype and Function in Myalgic Encephalomyelitis/Chronic Fatigue Syndrome Patients
Source: Front Immunol. 2017 Jun 26;8:723. doi: 10.3389/fimmu.2017.00723 (PMC5483846; doi:10.3389/fimmu.2017.00723)
Supplement: Supplementary file 5 [file Presentation_1.PDF]

Supplementary methods

Clinical questionnaires

Four scales showing the clinical phenotype of the ME/CFS patients were used. Hospital Anxiety and Depression scale (HADS) was used to determine the levels of anxiety and depression that the study participants were experiencing (Zigmond and Snaith 1983). The HADS anxiety and HADS depression scores range from 0 to 21. A score of equal or more than 10 allows for clinically significant anxiety or depression symptoms to be suspected.

The Short-Form 36 (SF-36) is a validated and widely used questionnaire to assess health-related quality of life (Sullivan, Karlsson, and Ware 1995; Sullivan and Karlsson 1998). The 36 items in the questionnaire are grouped into 8 subscale scores listed in Supplementary Table 1. The subscale scores range from 0 to 100, with a higher score indicating a better health-related quality of life.

In order to directly study ME/CFS symptoms, patients were asked to rate the occurrence and severity of the common symptoms listed in Supplementary Table 1. Symptom severity during the last week was rated on a 0-4-point scale ("none", "mild", "moderate", "severe", "unbearable"). This questionnaire has previously been used to study clinical subgroups (Jonsjö et al. 2017).

For statistical comparisons of the patient groups from Stockholm and Oslo, the same procedure was used as for the laboratory data. In short, for variables with normal distribution before or after log10 transformation, Students T-test was used. For non-normal variables, Mann-Whitney U test was used.

Phenotypic stainings

For the studies of cell quantities in peripheral blood, blood was collected in heparin tubes and stained with fluorochrome-conjugated antibodies as indicated in Table S2 in Trucount tubes (BD biosciences) within 6 hours of venepuncture. After 15 minutes, the erythrocytes were lysed using a lysis solution for 15 minutes (BD biosciences). This was followed by flow cytometry.

For intracellular stainings of perforin, granzymes as well as base-line CD69 and activation panel for Oslo, freshly thawed PBMC were surface stained with fluorochrome-conjugated antibodies in FACS buffer for 30 minutes at room temperature (see Table S2 for a list of antibodies). Cells were then fixed in a paraformaldehyde-containing buffer (Fix/Perm Buffer, BD Biosciences) followed by intracellular staining with antibodies indicated in Table S2 for 30 minutes at room temperature with a saponin containing permeabilization solution (Perm/Wash Buffer, BD Biosciences). As controls for the internal stainings, a set of isotype control antibodies was used. These stainings were followed by analyses by flow cytometry.

For cytotoxic lymphocyte activation and adaptive NK cell panels, freshly thawed PBMC were surface stained with fluorochrome-conjugated antibodies as indicated in Table S2 for 30 minutes at room temperature in FACS buffer. Cells were then fixed in a paraformaldehyde containing buffer (Fix/Perm Buffer, BD Biosciences) at 37°C for 15 minutes. After this, the cells were permeabilized in PBS supplemented with 0.05% Triton X-100 (Sigma-Aldrich) for 5 minutes at room temperature, followed by primary (and for the adaptive NK cell panel, secondary and tertiary) intracellular stainings (Table S2), which were performed for 30 minutes at room temperature (the tertiary intracellular staining for the adaptive NK cell panel was prolonged to two hours) in FACS buffer.

The gating strategies for the different panels were similar, as outlined (Fig. 4A). First, lymphocytes and single cells were identified with forward scatter/side scatter

characteristics. Thereafter, cells negative for CD4, CD14, CD19 ($CD20^{bright}$ in the true count panel) and negative for the dead cell marker were selected. This was followed by a division into CD3 positive and negative cells. In the CD3 negative compartment, $CD56^{dim}$ and $CD56^{bright}$ cells were separated, aided by the exclusive CD57 expression by the $CD56^{dim}$ cells. In the CD3 positive compartment, cells negative for TCR-γδ (in all panels except the TruCount panel) and positive for CD8 were identified. These cells were further divided into CD57 positive and CD57 negative cells.

For the TruCount panel, counts were calculated based on the relation in cell subset number to the number of acquired beads in the sample.

For the cytotoxic protein panels, median fluorescence values were calculated as median(x)-median(iso_x), where x a specific cell type in a specific donor and iso_x is the isotype control for the same cell type and donor. One exception was perforin in the Stockholm sample that was instead calculated as median(x)-median($CD4_x$) where x is a specific cell type in a specific donor and $CD4_x$ is the $CD3^+CD4^+CD57^-$ T cell population (that lacks perforin expression) in the same donor. The cytotoxic proteins in the $CD3^+CD8^+CD57^-$ compartment are bimodally expressed, due to the heterogeneity of this cell population. For these cells, the cytotoxic proteins were quantified using histogram gates instead.

For the activation and adaptive NK cell panels, gates were made on histograms for the markers of interest.

Functional evaluations using flow cytometry

For assessment of functional responses in the Stockholm sample, thawed PBMC that had been rested overnight were incubated complete media containing differing concentrations of CellTracker Green (Invitrogen, Waltham, MA) for 15 minutes, to bar code the samples for the final flow cytometry steps. Thereafter, the media was exchanged to complete media supplemented with Brefeldin A (GolgiPlug, BD Biosciences), or to complete media supplemented with Brefeldin A in combination with K562 cells, Raji cells, Raji cells supplemented with Rituximab antibody, P815 cells, P815 cells supplemented with anti-CD3, anti-CD16 or anti-CD226+anti-CD244. Following a 4-hour stimulation, the eight stimulated samples from each donor were surface stained with antibodies as indicated in Table S2 at room temperature in FACS buffer. During the 30-minute staining, four samples from each donor were concatenated in one well, resulting in two wells per donor. Cells were then fixed in Fix/Perm buffer followed by intracellular staining with antibodies to TNF and IFN-γ for 30 minutes at room temperature in Perm/Wash buffer. This was followed by flow cytometric analysis.

For the Oslo patients, the experiment was repeated in a similar fashion, but P815 cells, P815 cells supplemented with anti-CD3, anti-CD16 and anti-CD226+anti-CD244 were the targets. The panel was also adjusted. See Table S2. Erroneously, the UCHT.1 clone of anti-CD3 antibody was used for stimulation instead of S4.1, that was used in the Stockholm cohort. This likely explains the weaker responses in the Oslo $CD8^+$ T cell subsets.

For assessment of functional responses after inhibition with adrenaline, PBMC that had been rested overnight were bar coded as described above, and then complete media containing 1.1, 0.11 0.011 or 0 μM adrenaline was added and the cells were incubated for 15 minutes at 37°C in a humified incubator with 5% CO_2. Thereafter, stimulation solutions based on complete media supplemented with Brefeldin A and added P815 cells or P815 cells with anti-CD16 was added. Following a 4-hour stimulation, the samples from each donor were surface stained with antibodies as

indicated in Table S2 at room temperature in FACS buffer. During the 30-minute staining, the two samples from each donor and concentration were concatenated. Cells were then fixed in Fix/Perm buffer followed by intracellular staining with antibodies to TNF and IFN-γ for 30 minutes at room temperature in Perm/Wash buffer. After this, flow cytometry was performed.

The general gating strategy for the functional experiments is outlined in fig. S1. First, lymphocytes and single cells were identified with forward scatter/side scatter characteristics. This was followed by identification of $CD14^{-}CD19^{-}$ living cells, and separation of $CD3^{+}$ and $CD3^{-}$ cells. In the CD3+ compartment, TCR-$\gamma\delta^{+}$ T-cells were excluded, followed by identification of $CD4^{-}CD8^{+}$ cells. After this, CD57+ and CD57- cells were separated. In the CD3- compartment, CD56bright and dim cells were identified. As the different stimulations had different intensities of the barcode, the remaining cell populations for the four CD8 T and NK cell subsets were separated into different stimulations (in the follow-up Oslo experiment, no bar code was included). Gates for CD107a, IFN-γ and TNF were subsequently drawn based on the most relevant non-stimulatory condition, *i.e.* no target or P815.

Cytotoxicity assay

For assessment of cytotoxicity by PBMC, the cells were thawed and rested overnight in complete media. PBMC at different concentrations were mixed with 5×10^{3} ^{51}Cr-labeled K562 cells and incubated in duplicate for 4 hours at 37°C. Effector-to-target cell ratios ranged from 10 to 0.3 in 96-well V-bottom plates. The supernatants were measured for ^{51}Cr-release on a gamma-counter (Perklin-Elmer, Waltham, MA). Cytotoxic activity (specific NK cell lysis) in samples was measured with the following formula: (x-min)/(max-min)/(NK cell fraction), where 'x' is the average value from the doublets acquired for every sample, 'min' is the spontaneous ^{51}Cr release from K562 cells, 'max' is the ^{51}Cr release from target cells lysed in 1M HCl, and 'NK cell fraction' is the fraction of NK cells out of total lymphocytes from the same donor in the phenotypic stainings performed simultaneously.

Statistical methods

Probing for normal distributions, subsequent transformations, Student T test and Mann-Whitney U test

For all investigated cytotoxic lymphocyte phenotypic and functional read-outs, the Shapiro-Wilk (Shapiro and Wilk 1965) test was used to test if normal distribution could be assumed. In the cases where this was not the case, a log10-transformation was performed and the Shapiro-Wilk test was re-performed.

For Fig. 1-5, if normality could not be assumed even after transformation, p-values were obtained with the non-parametric Mann-Whitney U test. In all other cases, a Student T test was performed to compare the groups.

For the principal component, sparse projection to latent structures discriminant, receiver operating characteristic and Pearson product-moment correlation analyses, where normality was a prerequisite, parameters where the log10-transformation did not result in normality were first reversed by taking max($parameter_x$+1)-x where x is any value and max($parameter_x$) is the highest value in the parameter where x is present, and subsequently log10 transformed. After this, the Shapiro-Wilk test was re-performed and the distribution amongst the original, the log10 transformed and the rev-log10 transformed version of the data that was closest to normality was selected. Scripts were written in R (version 3.2 (R Core Team 2017)) for this, and included the use of package fBasics (Rmetrics Core Team et al. 2014).

SPICE analysis of adaptive NK cell subsets

For the analysis of all possible combinations of NKG2C, EAT-2, FcER1γ, SYK and PLZF in the Oslo sample, SPICE (Roederer, Nozzi, and Nason 2011) was deployed. First, Boolean gating was performed using FlowJo, and the percentage of cells being positive for any combination of markers was exported. This information was then pre-processed to a SPICE-compatible format in Excel and then imported to SPICE. In SPICE, the donors were grouped, and the ME/CFS patients and controls were overlaid, whereas NKG2C, EAT-2, FcER1γ, SYK and PLZF were categorical.

Principal component analysis

Principal component analysis (PCA) was performed on the 51 variables common to the Stockholm and the Oslo analysis. The pre-processing steps were transformation to obtain normal distribution (see above for details), followed by mean centering and unit variance scaling. A PCA model with 51 components was developed for Stockholm and 43 components for Oslo (due to a total number of 43 donors), 14 components were chosen. These together explained ≈80% of the variance (Fig. S2a). For this, R package mixOmics (Cao et al. 2016) was used. For visualization, R packages base (R Core Team 2017) and ggplot2 (Hadley 2009) was used.

Sparse projection to latent structures discriminant analysis

Projection to latent structures discriminant analysis (PLS-DA) is a supervised method that identifies the angle through a multidimensional data cloud that separates two groups at maximum. Methods based on projection to latent structures are very useful for data analysis where there are a number of correlated measurements and where the number of observations is lower than or equal to the number of measurements. Sparse PLS-DA (sPLS-DA) is a variant of PLS-DA that identify the original measurements that contribute to angle through the data cloud that separates the groups (Lê Cao, Boitard, and Besse 2011).

Before the sPLS-DA analyses, the data was transformed to obtain normal distribution, followed by mean centering and unit variance scaling. To increase the generalizability of the findings, the sPLS-DA performed on 1000 bootstrap subsamples, each with five cross-validation folds, using the R package bootsPLS (Rohart, Cao, and Wells 2015). In accordance with previous studies, one-dimensional models were created (Lê Cao, Boitard, and Besse 2011). The result was visualized with r package ggplot2 (Hadley 2009).

Cross-over prediction modelling using sPLS-DA result with receiver operating characteristic (ROC) curves

ROC curves were generated by taking the variables and loadings from the sPLS-DA in cohort 1 (*e.g.* Stockholm), retrieve the same variables from cohort 2 (*e.g.* Oslo), transform them in the same way as the original variables (from cohort 1) and then multiply them by the sPLS-DA loadings from cohort 1 to synthesize the same component/vector as in the cohort 1 sPLS-DA. This component/vector was then used as input in the ROC analysis for cohort 2 that was performed with the R package pROC.

Comparison of clinical-laboratory correlations between the Stockholm and Oslo sample

All 51 cytotoxic lymphocyte values were transformed to their most normal distribution, as described above. Thereafter, the Pearson product moment-correlations between the 21 clinical and the transformed cytotoxic lymphocyte values were calculated using the cor.test function in the R stats package (R Core Team 2017). Both the correlation estimate and the correlation p-values were exported. Then, the correlation estimates from the Oslo sample were plotted as a function of the correlation estimates from the Stockholm group. P-values lower than 0.01 are highlightet with with colors.

Citations in supplementary tables

For Supplementary table 1, see (Bjerkeset et al. 2008; Loge and Kaasa 1998).

For Supplementary Table 4, see (Klimas et al. 1990; See and Tilles 1996; Mawle et al. 1997; See et al. 1997; Ogawa et al. 1998; See et al. 1998; Masuda et al. 2002; Maher, Klimas, and Fletcher 2005; Fletcher et al. 2010; Ekua W. Brenu et al. 2010, 2011, 2012; Ekua Weba Brenu et al. 2014; Zhang et al. 1999; Stewart et al. 2003; Robertson et al. 2005; Curriu et al. 2013; Hardcastle et al. 2015; Huth et al. 2016).

Supplementary references

Bjerkeset, Ottar, Hans M. Nordahl, Sara Larsson, Alv A. Dahl, and Olav Linaker. 2008. "A 4-Year Follow-up Study of Syndromal and Sub-Syndromal Anxiety and Depression Symptoms in the General Population: The HUNT Study." *Social Psychiatry and Psychiatric Epidemiology* 43 (3): 192–99. doi:10.1007/s00127-007-0289-6.

Brenu, Ekua W., Mieke L. van Driel, Don R. Staines, Kevin J. Ashton, Sandra B. Ramos, James Keane, Nancy G. Klimas, and Sonya M. Marshall-Gradisnik. 2011. "Immunological Abnormalities as Potential Biomarkers in Chronic Fatigue Syndrome/Myalgic Encephalomyelitis." *Journal of Translational Medicine* 9 (May): 81. doi:10.1186/1479-5876-9-81.

Brenu, Ekua W., Mieke L. van Driel, Donald R. Staines, Kevin J. Ashton, Sharni L. Hardcastle, James Keane, Lotti Tajouri, Daniel Peterson, Sandra B. Ramos, and Sonya M. Marshall-Gradisnik. 2012. "Longitudinal Investigation of Natural Killer Cells and Cytokines in Chronic Fatigue Syndrome/Myalgic Encephalomyelitis." *Journal of Translational Medicine* 10 (May): 88. doi:10.1186/1479-5876-10-88.

Brenu, Ekua W., Donald R. Staines, Oguz K. Baskurt, Kevin J. Ashton, Sandra B. Ramos, Rhys M. Christy, and Sonya M. Marshall-Gradisnik. 2010. "Immune and Hemorheological Changes in Chronic Fatigue Syndrome." *Journal of Translational Medicine* 8 (January): 1. doi:10.1186/1479-5876-8-1.

Brenu, Ekua Weba, Teilah K. Huth, Sharni L. Hardcastle, Kirsty Fuller, Manprit Kaur, Samantha Johnston, Sandra B. Ramos, Don R. Staines, and Sonya M. Marshall-Gradisnik. 2014. "Role of Adaptive and Innate Immune Cells in Chronic Fatigue Syndrome/Myalgic Encephalomyelitis." *International Immunology* 26 (4): 233–42. doi:10.1093/intimm/dxt068.

Cao, Kim-Anh Le, Florian Rohart, Ignacio Gonzalez, Sebastien Dejean with key contributors Benoit Gautier, Francois Bartolo and contributions from Pierre Monget, Jeff Coquery, FangZou Yao, and Benoit Liquet. 2016. *mixOmics: Omics Data Integration Project* (version 6.1.1). https://cran.r-project.org/web/packages/mixOmics/index.html.

Curriu, Marta, Jorge Carrillo, Marta Massanella, Josepa Rigau, José Alegre, Jordi Puig, Ana M. Garcia-Quintana, et al. 2013. "Screening NK-, B- and T-Cell Phenotype and Function in Patients Suffering from Chronic Fatigue Syndrome." *Journal of Translational Medicine* 11 (March): 68. doi:10.1186/1479-5876-11-68.

Fletcher, Mary A., Xiao R. Zeng, Kevin Maher, Silvina Levis, Barry Hurwitz, Michael Antoni, Gordon Broderick, and Nancy G. Klimas. 2010. "Biomarkers in Chronic Fatigue Syndrome: Evaluation of Natural Killer Cell Function and Dipeptidyl Peptidase IV/CD26." *PloS One* 5 (5): e10817. doi:10.1371/journal.pone.0010817.

Hadley, Wickham. 2009. *ggplot2: Elegant Graphics for Data Analysis*. Springer-Verlag New York. http://ggplot2.org.

Hardcastle, Sharni Lee, Ekua Weba Brenu, Samantha Johnston, Thao Nguyen, Teilah Huth, Naomi Wong, Sandra Ramos, Donald Staines, and Sonya Marshall-Gradisnik. 2015. "Characterisation of Cell Functions and Receptors in Chronic Fatigue Syndrome/Myalgic Encephalomyelitis (CFS/ME)." *BMC Immunology* 16 (June): 35. doi:10.1186/s12865-015-0101-4.

Huth, T. K., E. W. Brenu, S. Ramos, T. Nguyen, S. Broadley, D. Staines, and S. Marshall-Gradisnik. 2016. “Pilot Study of Natural Killer Cells in Chronic Fatigue Syndrome/Myalgic Encephalomyelitis and Multiple Sclerosis.” *Scandinavian Journal of Immunology* 83 (1): 44–51. doi:10.1111/sji.12388.

Jonsjö, Martin A., Rikard K. Wicksell, Linda Holmström, Anna Andreasson, Indre Bileviciute-Ljungar, and Gunnar L. Olsson. 2017. “Identifying Symptom Subgroups in Patients with ME/CFS – Relationships to Functioning and Quality of Life.” *Fatigue: Biomedicine, Health & Behavior* 5 (1): 33–42. doi:10.1080/21641846.2017.1287546.

Klimas, N. G., F. R. Salvato, R. Morgan, and M. A. Fletcher. 1990. “Immunologic Abnormalities in Chronic Fatigue Syndrome.” *Journal of Clinical Microbiology* 28 (6): 1403–10.

Lê Cao, Kim-Anh, Simon Boitard, and Philippe Besse. 2011. “Sparse PLS Discriminant Analysis: Biologically Relevant Feature Selection and Graphical Displays for Multiclass Problems.” *BMC Bioinformatics* 12 (June): 253. doi:10.1186/1471-2105-12-253.

Loge, J. H., and S. Kaasa. 1998. “Short Form 36 (SF-36) Health Survey: Normative Data from the General Norwegian Population.” *Scandinavian Journal of Social Medicine* 26 (4): 250–58.

Maher, K. J., N. G. Klimas, and M. A. Fletcher. 2005. “Chronic Fatigue Syndrome Is Associated with Diminished Intracellular Perforin.” *Clinical and Experimental Immunology* 142 (3): 505–11. doi:10.1111/j.1365-2249.2005.02935.x.

Masuda, Akinori, Takao Munemoto, Takao Yamanaka, Michiko Takei, and Chuwa Tei. 2002. “Psychosocial Characteristics and Immunological Functions in Patients with Postinfectious Chronic Fatigue Syndrome and Noninfectious Chronic Fatigue Syndrome.” *Journal of Behavioral Medicine* 25 (5): 477–85.

Mawle, A. C., R. Nisenbaum, J. G. Dobbins, H. E. Gary, J. A. Stewart, M. Reyes, L. Steele, D. S. Schmid, and W. C. Reeves. 1997. “Immune Responses Associated with Chronic Fatigue Syndrome: A Case-Control Study.” *The Journal of Infectious Diseases* 175 (1): 136–41.

Ogawa, M., T. Nishiura, M. Yoshimura, Y. Horikawa, H. Yoshida, Y. Okajima, I. Matsumura, et al. 1998. “Decreased Nitric Oxide-Mediated Natural Killer Cell Activation in Chronic Fatigue Syndrome.” *European Journal of Clinical Investigation* 28 (11): 937–43.

R Core Team. 2017. *R: A Language and Environment for Statistical Computing*. Vienna, Austria: R Foundation for Statistical Computing. https://www.R-project.org.

Rmetrics Core Team, Diethelm Wuertz, Tobias Setz, and Yohan Chalabi. 2014. *fBasics: Rmetrics - Markets and Basic Statistics* (version 3011.87). https://cran.r-project.org/web/packages/fBasics/index.html.

Robertson, M. J., R. S. Schacterle, G. A. Mackin, S. N. Wilson, K. L. Bloomingdale, J. Ritz, and A. L. Komaroff. 2005. “Lymphocyte Subset Differences in Patients with Chronic Fatigue Syndrome, Multiple Sclerosis and Major Depression.” *Clinical and Experimental Immunology* 141 (2): 326–32. doi:10.1111/j.1365-2249.2005.02833.x.

Roederer, Mario, Joshua L. Nozzi, and Martha C. Nason. 2011. “SPICE: Exploration and Analysis of Post-Cytometric Complex Multivariate Datasets.” *Cytometry. Part A: The Journal of the International Society for Analytical Cytology* 79 (2): 167–74. doi:10.1002/cyto.a.21015.

Rohart, Florian, Kim-Anh Le Cao, and Christine Wells. 2015. *bootsPLS: Bootstrap Subsamplings of Sparse Partial Least Squares - Discriminant Analysis for Classification and Signature Identification* (version 1.0.3). https://cran.r-project.org/web/packages/bootsPLS/index.html.

See, D. M., N. Broumand, L. Sahl, and J. G. Tilles. 1997. “In Vitro Effects of Echinacea and Ginseng on Natural Killer and Antibody-Dependent Cell Cytotoxicity in Healthy Subjects and Chronic Fatigue Syndrome or Acquired Immunodeficiency Syndrome Patients.” *Immunopharmacology* 35 (3): 229–35.

See, D. M., P. Cimoch, S. Chou, J. Chang, and J. Tilles. 1998. “The in Vitro Immunomodulatory Effects of Glyconutrients on Peripheral Blood Mononuclear Cells of Patients with Chronic Fatigue Syndrome.” *Integrative Physiological and Behavioral Science: The Official Journal of the Pavlovian Society* 33 (3): 280–87.

See, D. M., and J. G. Tilles. 1996. “Alpha-Interferon Treatment of Patients with Chronic Fatigue Syndrome.” *Immunological Investigations* 25 (1–2): 153–64.

Shapiro, S. S., and M. B. Wilk. 1965. “An Analysis of Variance Test for Normality (Complete Samples).” *Biometrika* 52 (3–4): 591–611. doi:10.1093/biomet/52.3-4.591.

Stewart, Carleton C., Diane L. Cookfair, Kathleen M. Hovey, Karl E. Wende, David S. Bell, and Carolyn L. Warner. 2003. “Predictive Immunophenotypes: Disease-Related Profile in Chronic Fatigue Syndrome.” *Cytometry. Part B, Clinical Cytometry* 53 (1): 26–33. doi:10.1002/cyto.b.10034.

Sullivan, M., and J. Karlsson. 1998. “The Swedish SF-36 Health Survey III. Evaluation of Criterion-Based Validity: Results from Normative Population.” *Journal of Clinical Epidemiology* 51 (11): 1105–13.

Sullivan, M., J. Karlsson, and J. E. Ware. 1995. “The Swedish SF-36 Health Survey--I. Evaluation of Data Quality, Scaling Assumptions, Reliability and Construct Validity across General Populations in Sweden.” *Social Science & Medicine (1982)* 41 (10): 1349–58.

Zhang, Q., X. D. Zhou, T. Denny, J. E. Ottenweller, G. Lange, J. J. LaManca, M. H. Lavietes, C. Pollet, W. C. Gause, and B. H. Natelson. 1999. “Changes in Immune Parameters Seen in Gulf War Veterans but Not in Civilians with Chronic Fatigue Syndrome.” *Clinical and Diagnostic Laboratory Immunology* 6 (1): 6–13.

Zigmond, A. S., and R. P. Snaith. 1983. “The Hospital Anxiety and Depression Scale.” *Acta Psychiatrica Scandinavica* 67 (6): 361–70.

Supplementary figures and figure legends

Figure S1

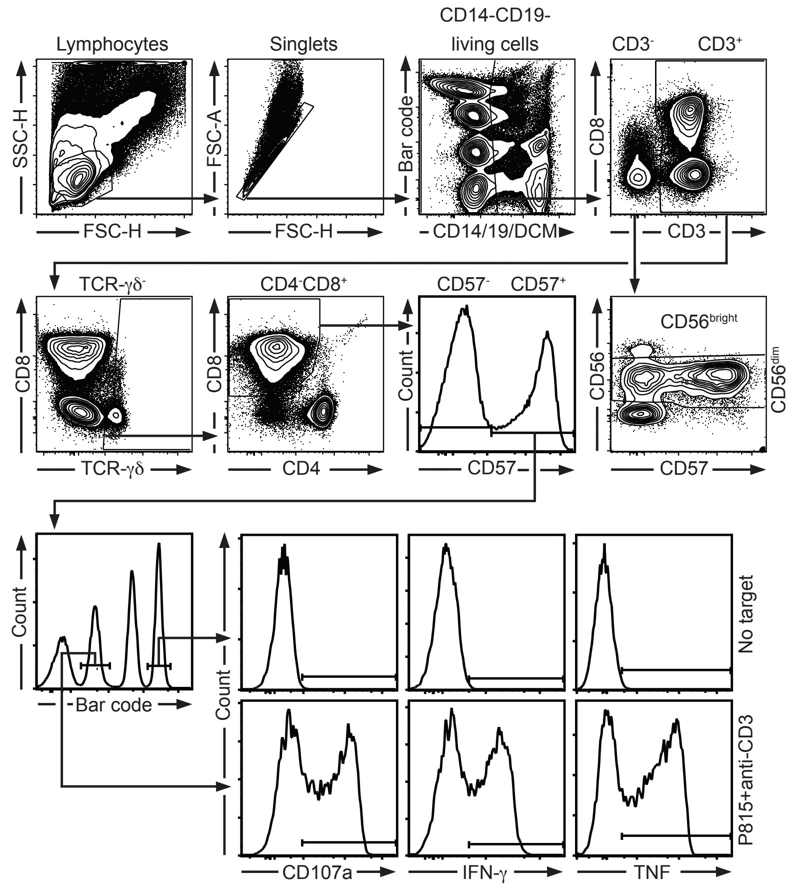

Gating strategy for functional panels. The full gating strategy is shown up to the four CD8+ T and NK cell subsets. For the $CD8^{+}CD57^{+}$ cells, the bar code and functional parameter gates are also included. DCM=dead cell marker. Bar code=cell tracker green at different concentrations. The data shows one representative donor.

Figure S2

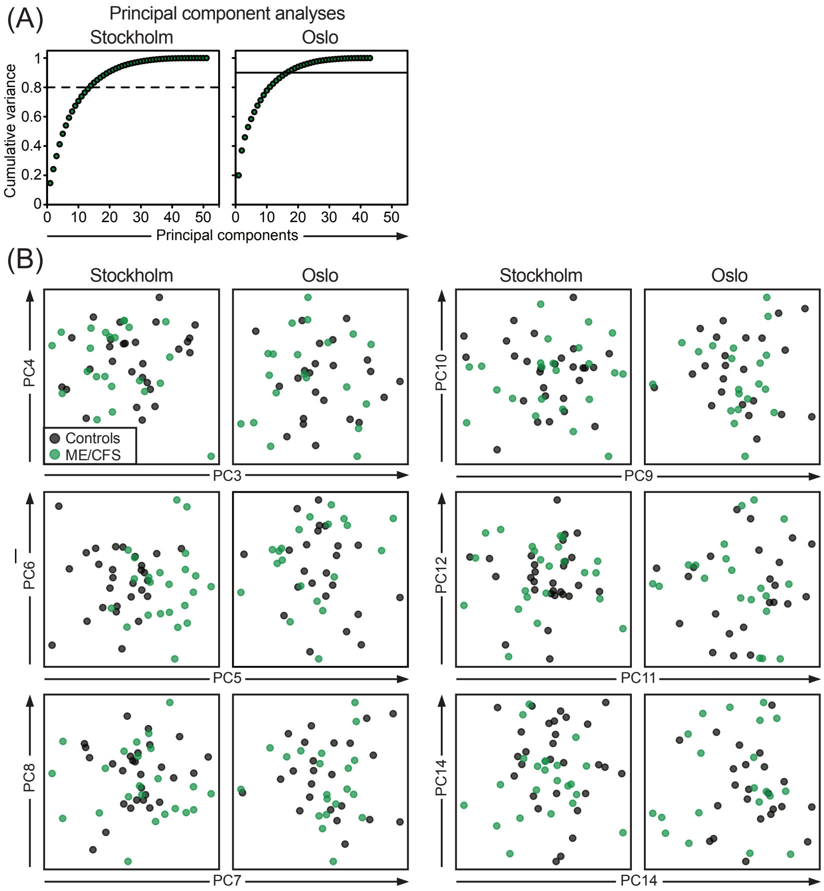

Principal component analyses for cytotoxic lymphocyte parameters common to the Stockholm and Oslo samples. Two columns are shown, where column one and two depicts separate analyses for the Stockholm and Oslo sample, respectively. (a): The cumulative percent of variance explained by the 51/43 principal components. (b): The distribution of the patients and controls in the 3rd to 14th principal component. Grey and black color indicates control and ME/CFS individual, respectively. For the Stockholm and Oslo samples respectively, 23+28, and 20+23 patients+controls, are included in the analyses.

Figure S3

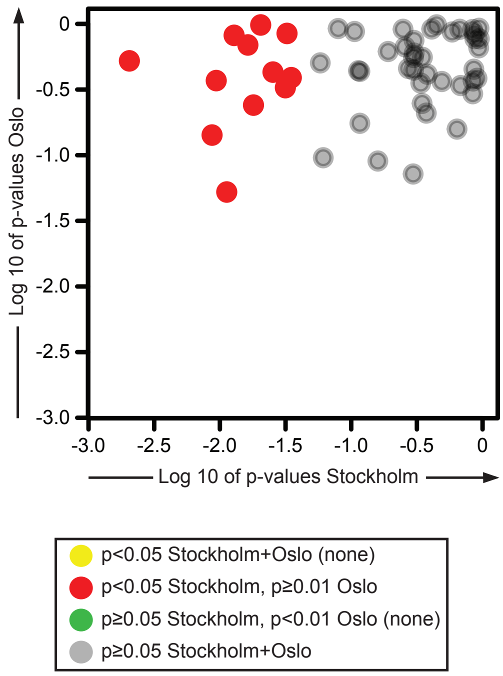

Comparison of p-values between the Stockholm and Oslo samples
Oslo p-values from single cytotoxic lymphocyte phenotypic and functional analyses are plotted as a function of Stockholm p-values from the same analyses. Yellow dots should show significant differences that overlap between the analyses (none present). Red and green dots should show significant differences in the Stockholm and Oslo sample, respectively (no green dots present).
